# *Vegfa* promoter gene hypermethylation at HIF1α binding site is an early contributor to CKD progression after renal ischemia

**DOI:** 10.1038/s41598-021-88000-5

**Published:** 2021-04-22

**Authors:** Andrea Sánchez-Navarro, Rosalba Pérez-Villalva, Adrián Rafael Murillo-de-Ozores, Miguel Ángel Martínez-Rojas, Jesús Rafael Rodríguez‐Aguilera, Norma González, María Castañeda-Bueno, Gerardo Gamba, Félix Recillas-Targa, Norma A. Bobadilla

**Affiliations:** 1 Molecular Physiology Unit, Instituto de Investigaciones Biomédicas, Universidad Nacional Autónoma de México, Av. Universidad 3000, UNAM, CU, 04510 Coyoacán, Mexico City, Mexico; 2 Department of Nephrology, Instituto Nacional de Ciencias Médicas y Nutrición Salvador Zubirán, Vasco de Quiroga No. 15, Tlalpan 14080, Mexico City, Mexico; 3 Department of Molecular Genetics, Instituto de Fisiología Celular, Universidad Nacional Autónoma de México, Mexico City, Mexico

**Keywords:** Physiology, Nephrology, Pathogenesis

## Abstract

Chronic hypoxia is a major contributor to Chronic Kidney Disease (CKD) after Acute Kidney Injury (AKI). However, the temporal relation between the acute insult and maladaptive renal response to hypoxia remains unclear. In this study, we analyzed the time-course of renal hemodynamics, oxidative stress, inflammation, and fibrosis, as well as epigenetic modifications, with focus on HIF1α/VEGF signaling, in the AKI to CKD transition. Sham-operated, right nephrectomy (UNx), and UNx plus renal ischemia (IR + UNx) groups of rats were included and studied at 1, 2, 3, or 4 months. The IR + UNx group developed CKD characterized by progressive proteinuria, renal dysfunction, tubular proliferation, and fibrosis. At first month post-ischemia, there was a twofold significant increase in oxidative stress and reduction in global DNA methylation that was maintained throughout the study. *Hif1α* and *Vegfa* expression were depressed in the first and second-months post-ischemia, and then *Hif1α* but not *Vegfa* expression was recovered. Interestingly, hypermethylation of the *Vegfa* promoter gene at the HIF1α binding site was found, since early stages of the CKD progression. Our findings suggest that renal hypoperfusion, inefficient hypoxic response, increased oxidative stress, DNA hypomethylation, and, *Vegfa* promoter gene hypermethylation at HIF1α binding site, are early determinants of AKI-to-CKD transition.

## Introduction

Clinical, epidemiological, and experimental studies have shown that AKI is an independent risk factor for the development of CKD and end-stage renal disease (ESRD)^1,2^. In this transition, the initial insult severity and duration is proportional to the risk of CKD, besides, age is another preponderant factor^3–6^.

AKI is characterized by an abrupt reduction in renal blood flow with consequent hypoxia, endothelial and proximal epithelial injury, and renal dysfunction. After an AKI episode, a cascade of events occurs, such as brush border loss, cell polarity alterations, increased oxidative stress, and mitochondrial dysfunction of proximal tubular epithelial cells^7,8^, as a result, some of these cells undergo necrosis or apoptosis^9^. These processes are also accompanied by macrophage infiltration and inflammation^7,10,11^. Although the tubular epithelium has the capacity for regeneration^12^, the injured epithelium is no longer the same. A subpopulation of dedifferentiated and proliferating tubular cells that recover from the acute renal insult suffer cell cycle arrest and cannot be re-differentiated, leading to tubular atrophy; all these events contribute greatly to the tubulointerstitial fibrosis that is observed in the long term^12,13^.

Consequently, a maladaptive repair occurs^11,14,15^, where there is persistent macrophage infiltration^16–18^, dissociation of pericytes from the tubular capillaries^19^, and arrest of some tubular cells in the G2/M phase^20^, leading to a progressive fibrotic kidney^20–22^. Some mechanisms involved have been elucidated, such as trans-differentiation of pericytes into myofibroblasts^19,23^, uncontrolled proliferation of epithelial cells^22^, the emergence of an abnormal proximal-tubule phenotype^24^, excessive production of TGFβ by both the tubular epithelium^22^ and local myofibroblasts^25,26^, accumulation of extracellular matrix proteins^27,28^, vascular rarefaction^29–31^, chronic hypoxia^30,32^, and chronic stress of the endoplasmic reticulum^33^.

In addition, it has been recently shown that epigenetic modifications may be involved in several renal pathologies. However, the specific molecular mechanisms by which epigenetic modifications alter renal physiology are little known. The most studied epigenetic regulations in AKI are the chromatin compaction, DNA methylation and histone acetylation/deacetylation. In AKI, obstructive renal injury, and diabetic nephropathy, epigenetic modifications induced an increase in proinflammatory and profibrotic cytokines such as monocyte chemoattractant protein-1 (MCP-1), complement protein 3 (C3), transforming growth factor β (TGF-β), which in turn perpetuate inflammation and promote epithelial-to-mesenchymal transition (EMT) that contributes to renal fibrosis^34–38^. Although some mechanisms have been elucidated, many others remain unknown, and less is known about temporal changes during CKD progression, such as renal hemodynamics, structural injury, HIF signaling, and epigenetic modifications.

We have previously shown that an AKI episode induced by moderate or severe bilateral renal ischemia/reperfusion (IR) in male rats is sufficient to induce CKD progression after nine months^22,27^. Interestingly, this transition was not observed in female rats, despite a similar magnitude of AKI in both male and female rats. The unique difference in the early phase post-ischemia was that females did not exhibit oxidative stress, suggesting a pivotal role of reactive oxygen species generation in this transition^21^.

Thus, it is relevant to establish a temporal understanding of the pathophysiological mechanisms throughout the AKI-to-CKD transition. In this study, we used the model of unilateral renal IR plus contralateral nephrectomy, which allowed us to induce CKD after four months. We found abnormal renal hemodynamics, reduced HIF-1α signaling, increased oxidative stress, and global DNA hypomethylation in the early phase of the AKI to CKD transition. We also showed that the *HIF-1α/Vegfa* signaling reduction was associated with the DNA hypermethylation of the *Vegfa* gene promoter, beginning at an early stage post-ischemia and suggesting that reduced VEGF expression is an early contributor that triggers renal hypoxia and the consequent fibrosis.

## Results

### Renal injury induced by unilateral ischemia after 24 h of reperfusion

**Figure 1:**
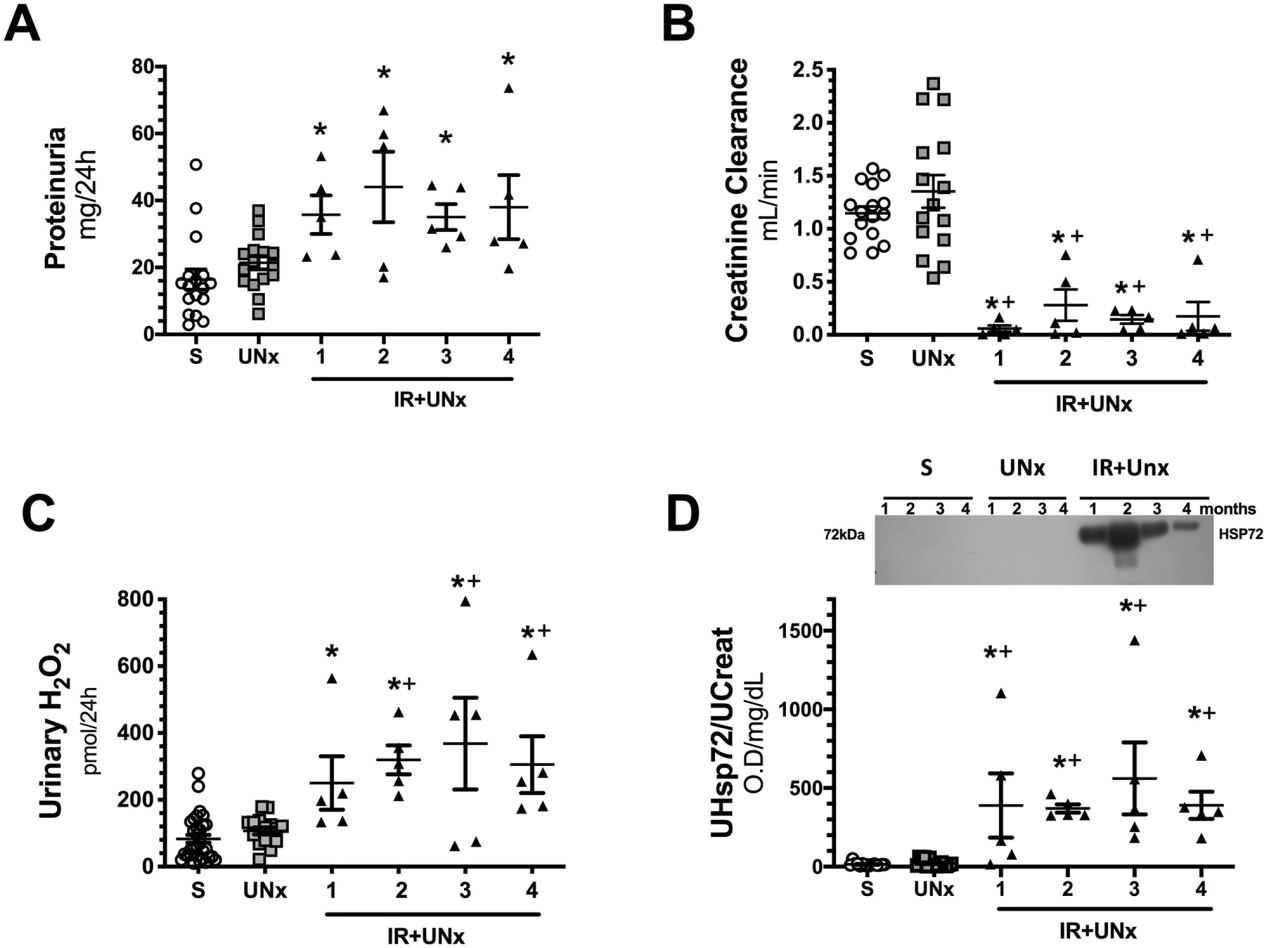
Renal injury induced by unilateral ischemia after 24 h of reperfusion. (**A**) Proteinuria, (**B**) Creatinine clearance, (**C**) Urinary hydrogen peroxide, and (**D**) Urinary HSP72 corrected by urinary creatinine, including a representative cropped blot image. Data are represented as the mean ± SE (for S, n = 16, for UNx, n = 16 and n = 20 for IR + UNx groups). White bars represent the S, gray bars represent UNx, and black bars represent IR + UNx groups. The one-way analysis of variance (ANOVA) was used to determine statistical differences, using the Bonferroni correction for multiple comparisons. *p < 0.05 versus S group and + *p* < 0.05 versus UNx group. Full-length blots are presented in Supplementary Fig. 3.

First, we corroborated that the initial insult induced by IR in the uninephrectomized rats was similar among the groups studied at 1, 2, 3, or 4 months postischemia. All IR + UNx rats were randomly assigned to the different periods of follow-up. After 24 h of inducing renal ischemia, all the IR + UNx rats exhibited significant proteinuria that was of the same magnitude among the groups assigned to 1, 2, 3 and, 4 months of follow-up (Fig. 1A), together with a similar reduction in renal function (Fig. 1B); these alterations were not observed in the S (n = 16) or UNx (n = 16) groups after 24 h of the surgery as is shown by the individual data presented in Fig. 1A, B. Consequently, the S or UNx rats were also randomly assigned to the different periods of follow-up. The urinary hydrogen peroxide levels were also evaluated and reflected significant oxidative stress in all IR + UNx groups (Fig. 1C). The urinary HSP72 levels, known to be a sensitive AKI biomarker, were also analyzed^39–42^. As expected, all rats that underwent IR + UNx exhibited a significant and similar increase in urinary HSP72 levels corrected by urinary creatinine (UHSP72/UCreat), (Fig. 1D). These findings show that all the IR + UNx rats exhibited a similar AKI degree. This was important to ensure that the changes observed in the long term were due to the initial insult itself rather than differences in the severity of the ischemic injury.

### Temporal progression of renal dysfunction and structural injury after an AKI episode

**Figure 2:**
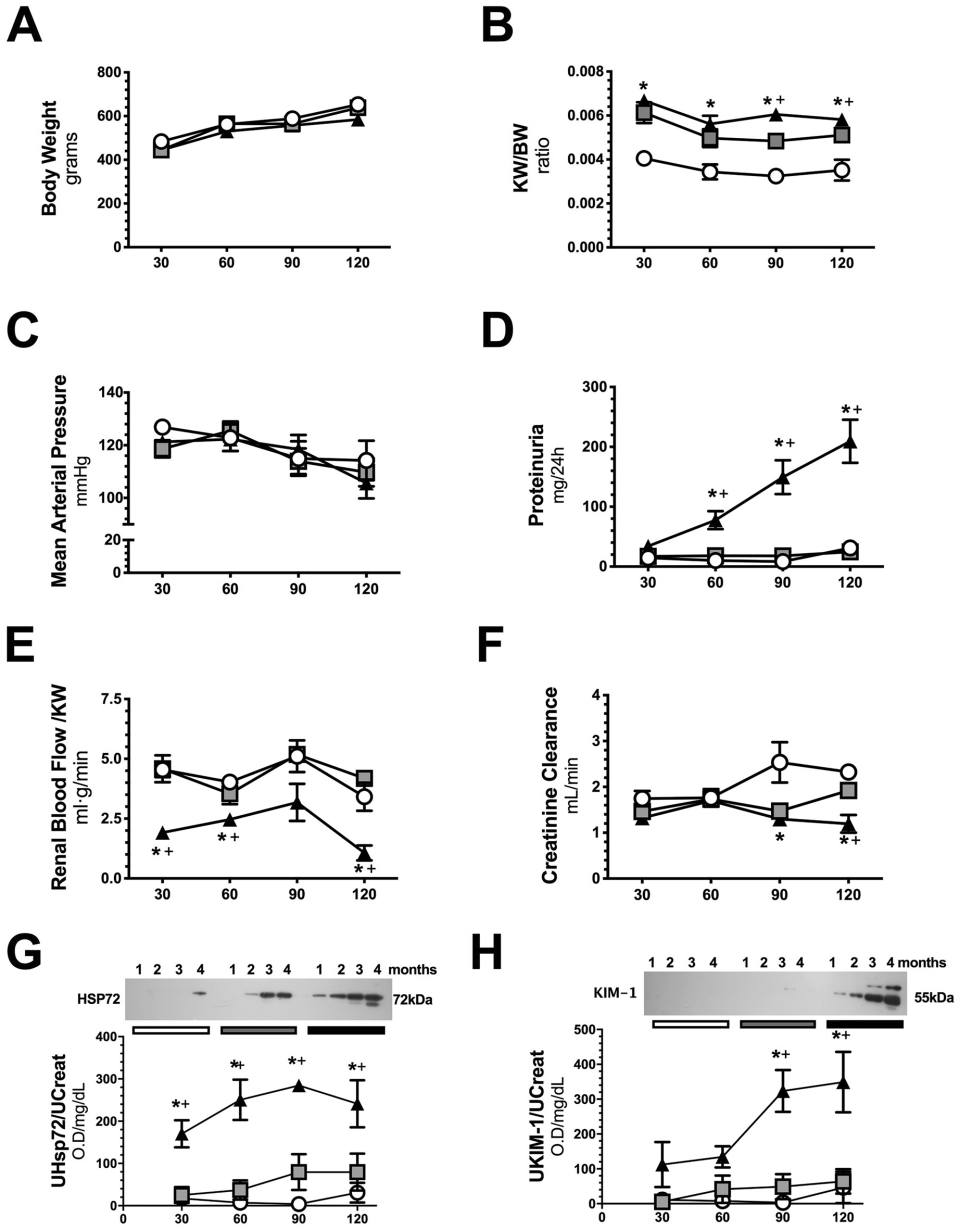
Follow-up of renal function in the AKI to CKD transition. (**A**) Body weight, (**B**) Ratio kidney weight/body weight, (**C**) Mean arterial Pressure, (**D**) Proteinuria, (**E**) Renal blood flow / kidney weight, (**F**) Creatinine clearance, (**G**) Urinary HSP72 corrected by urinary creatinine, and (**H**) Urinary KIM-1 corrected by urinary creatinine, including representative cropped blots, along the follow-up. Data are represented as the mean ± SE. n = 4, 4, and 5 for the S, UNx, and IR + UNx groups in each studied period: 30, 60, 90, and 120 days post-ischemia. White circles represent the S, gray squares represent UNx and black triangles represent IR + UNx groups. The one-way analysis of variance (ANOVA) was used to determine statistical differences, using the Bonferroni correction for multiple comparisons. *p < 0.05 versus S group and + *p* < 0.05 versus UNx group in their respective period. Full-length blots are presented in Supplementary Figs. 4 and 5.

To evaluate the precise moment at which the functional, structural, and molecular alterations occur along with AKI to CKD transition, the groups were euthanized at 1, 2, 3, or 4 months after the initial ischemic insult. No differences in body weight were found among the studied groups (Fig. 2A). As expected, the UNx and IR + UNx groups showed a significant increase in kidney weight/body weight (KW/BW) starting in the first-month compared to that of the S group (Fig. 2B). All experimental groups remained normotensive at the time of evaluation (Fig. 2C). The IR + UNx group exhibited a progressive increase in proteinuria starting in the second month of follow-up that was not evident in the S and UNx groups (Fig. 2D). Due to the renal mass reduction, the UNx group exhibited renal hyperperfusion, but when it was correct to the kidney weight, renal blood flow (RBF) was similar to the S group (Fig. 2E). This finding, in the UNx group, was related to the maintenance of normal renal function (Fig. 2F). Interestingly, this compensatory response was not seen in the IR + UNx group. RBF/KW was significantly lower than that of the S and UNx groups, starting in the first-month, and renal hypoperfusion was maintaining along the study course (Fig. 2E). At the end of the study, the IR + UNx group had significant renal dysfunction (Fig. 2F). The renal urinary biomarker HSP72 and KIM1 normalized by urinary creatinine, were significantly elevated starting in the first-month and third-month respectively, and increased even more by the end of the study (Fig. 2G, H). In Table 1 appears urinary flow, fractional excretion of sodium (FENa) and osmolarity. No differences were found among the groups along the study, except that the osmolarity was lower in the IR + UNx group at fourth-month compared to S group.

**Table 1:** Urine Chemistries along the study for all the included groups.

	Urinary flow (mL/min)	FENa (%)	Osmolarity (mOsm/L)
**Sham**			
30 days	0.031 ± 0.004	0.17 ± 0.03	350 ± 77
60 days	0.026 ± 0.007	0.10 ± 0.00	481 ± 107
90 days	0.023 ± 0.003	0.11 ± 0.02	729 ± 123
120 days	0.014 ± 0.003	0.08 ± 0.03	1226 ± 178
**UNx**			
30 days	0.029 ± 0.005	0.19 ± 0.04	494 ± 172
60 days	0.025 ± 0.005	0.15 ± 0.05	703 ± 119
90 days	0.014 ± 0.007	0.16 ± 0.04	936 ± 72
120 days	0.024 ± 0.004	0.09 ± 0.02	746 ± 129
**IR + UNx**			
30 days	0.026 ± 0.005	0.21 ± 0.07	448 ± 59
60 days	0.037 ± 0.003	0.26 ± 0.14	440 ± 39
90 days	0.030 ± 0.002	0.24 ± 0.07	501 ± 30
120 days	0.024 ± 0.004	0.20 ± 0.08	678 ± 145*

*p < 0.05 versus respective Sham.

**Figure 3:**
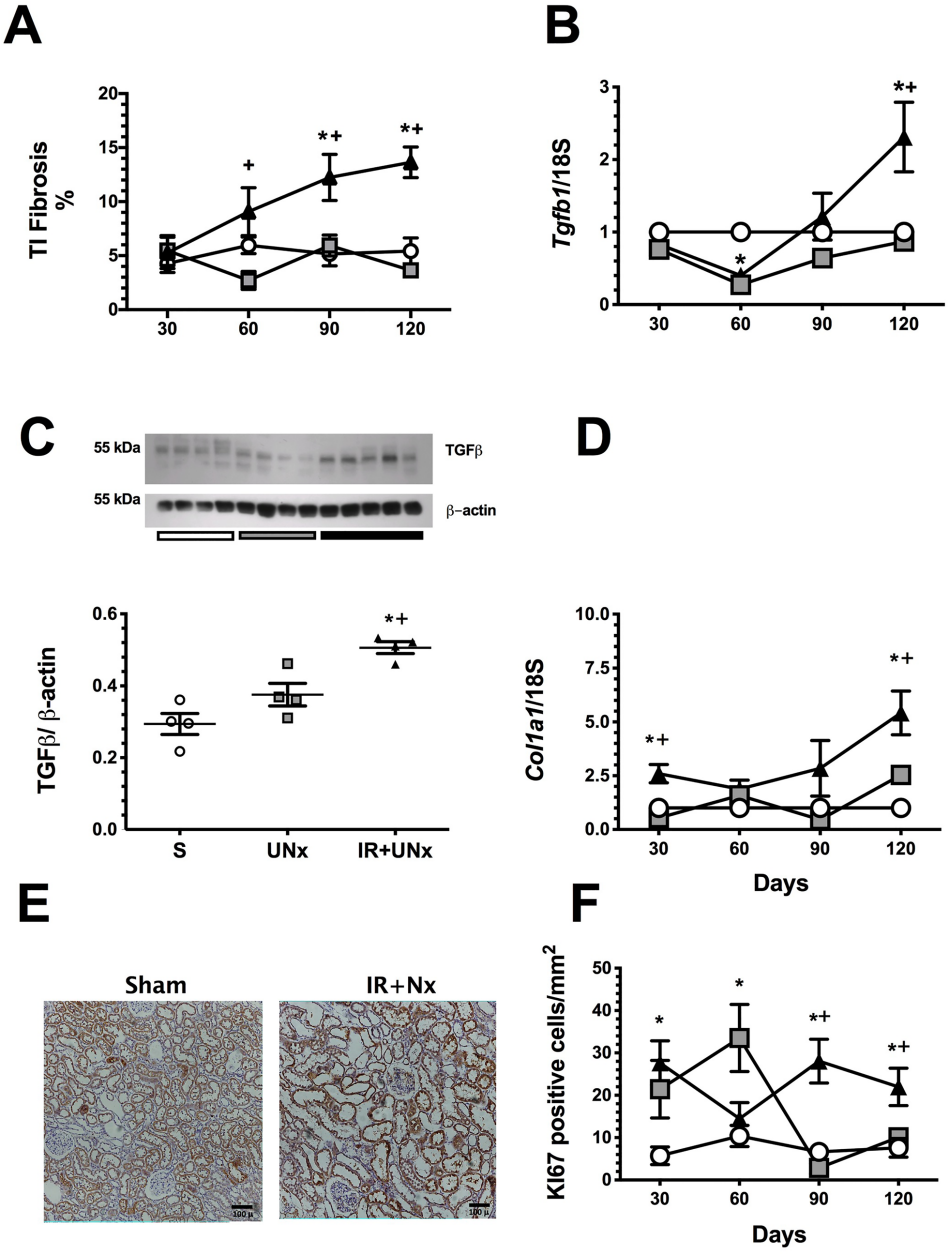
Temporally induction of tubulointersticial fibrosis by TGFβ activation. (**A**) Tubulointersticial fibrosis, (**B**) mRNA levels of *Tgfb1*, (**C**) Protein levels of TGFβ, at fourth-month post-ischemia, including a representative cropped blots. (**D**) mRNA levels of *Collagen1a1*, (**E**) Representative microphotographs of Ki67 immunostaining for the S and IR + UNx groups. (**F**) Quantification of Ki67 positive epithelial cells (cells/mm^2^). Data are represented as the mean ± SE. n = 4, 4, and 5 for S, UNx, and IR + UNx groups, in each studied period: 30, 60, 90, and 120 days post-ischemia. White circles/bar represent the S, gray squares/bar represent the UNx and black triangles/bar represent the IR + UNx groups. The one-way analysis of variance (ANOVA) was used to determine statistical differences, using the Bonferroni correction for multiple comparisons. *p < 0.05 versus S group and + *p* < 0.05 versus UNx group in their respective period. Full-length blots are presented in Supplementary Fig. 6.

The long-term consequences of an AKI episode were also evidenced by the presence of tubulointerstitial fibrosis starting in the second-month post-ischemia, which progressively increased, whereas this injury was not detected in the UNx group (Fig. 3A). Although increased *Tgfb1* mRNA levels were not observed in the early stages of the AKI to CKD transition, a significant upregulation in *Tgfb1* mRNA and protein levels was evident in the fourth-month post-ischemia compared to that of the S and UNx groups (Fig. 3B, C). Accordingly with this, *Col1a1* (collagen 1*)* mRNA levels were significantly increased in the fourth-month (Fig. 3D). We only measured TGFβ protein levels at the fourth-month, because the *Tgfb1* mRNA levels and its target gene *Col1a1* were only significantly increased in this point of the follow-up. Besides, the IR + UNx group exhibited higher levels of Ki67 positive tubular cells (Fig. 3E, F), similar to our previous findings^22^.

These results show that an AKI episode induced functional and structural alterations that mostly appear beginning at an early stage, highlighting the fact that there was no compensatory renal hyperperfusion expected by the renal mass lost in the IR + UNx group.

### Temporal course of oxidative stress and vasoactive factors in the CKD progression induced by an AKI episode

**Figure 4:**
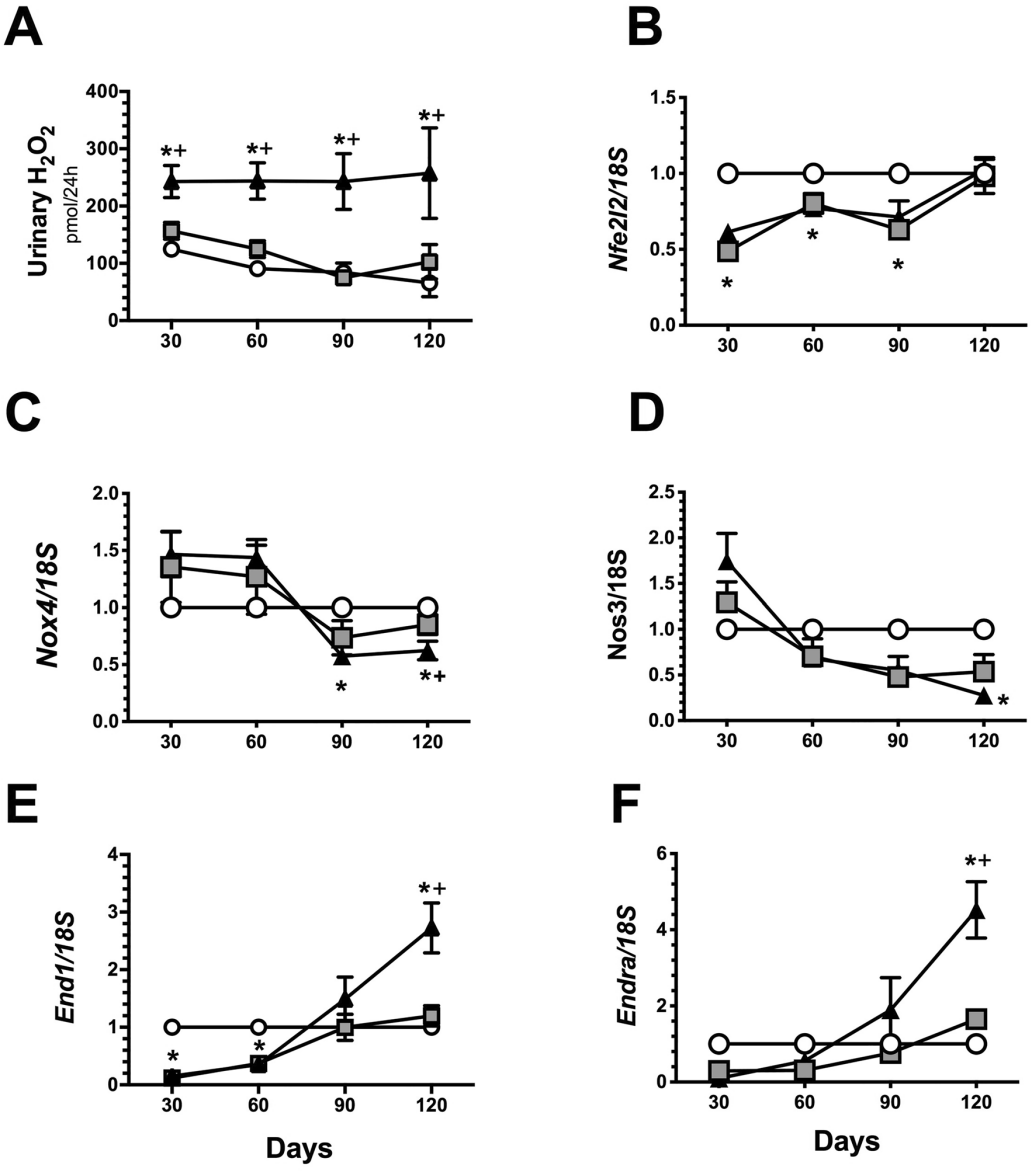
Oxidative stress and vasoactive mediators over the course of AKI to CKD transition. (**A**) Urinary hydrogen peroxide, (**B**) mRNA levels of *Nfe2l2* (**C**) mRNA levels of *Nos3,* (**D**) mRNA levels of NOX4, (**E**) mRNA levels of *Edn1* and (**F**) mRNA levels of endothelin receptor A (*Endra*). Data are represented as the mean ± SE. n = 4, n = 4, and n = 5 for the S, UNx, and IR + UNx groups, in each studied period: 30, 60, 90, and 120 days post-ischemia. The one-way analysis of variance (ANOVA) was used to determine statistical differences, using the Bonferroni correction for multiple comparisons. *p < 0.05 versus S group and + *p* < 0.05 versus UNx group in their respective period.

Oxidative stress and renal inflammation have a pivotal role in CKD progression; thus, the temporality of these two pathways was also analyzed. An increase in oxidative stress (Fig. 4A) and a reduction in mRNA levels of the transcription factor *Nfe2l2*, which stimulates the antioxidant response, was observed beginning in the initial stage of the AKI to CKD transition (Fig. 4B), even though *Nox4* mRNA levels were reduced in the late stage of the disease (Fig. 4C). An imbalance in vasoactive factors was also observed at the end of the study. *NOS3* mRNA levels were significantly decreased (Fig. 4D), whereas the endothelin vasoconstrictor effect was increased (Fig. 4E, F).

### Inflammatory pathways in the time course of CKD induced by AKI

**Figure 5:**
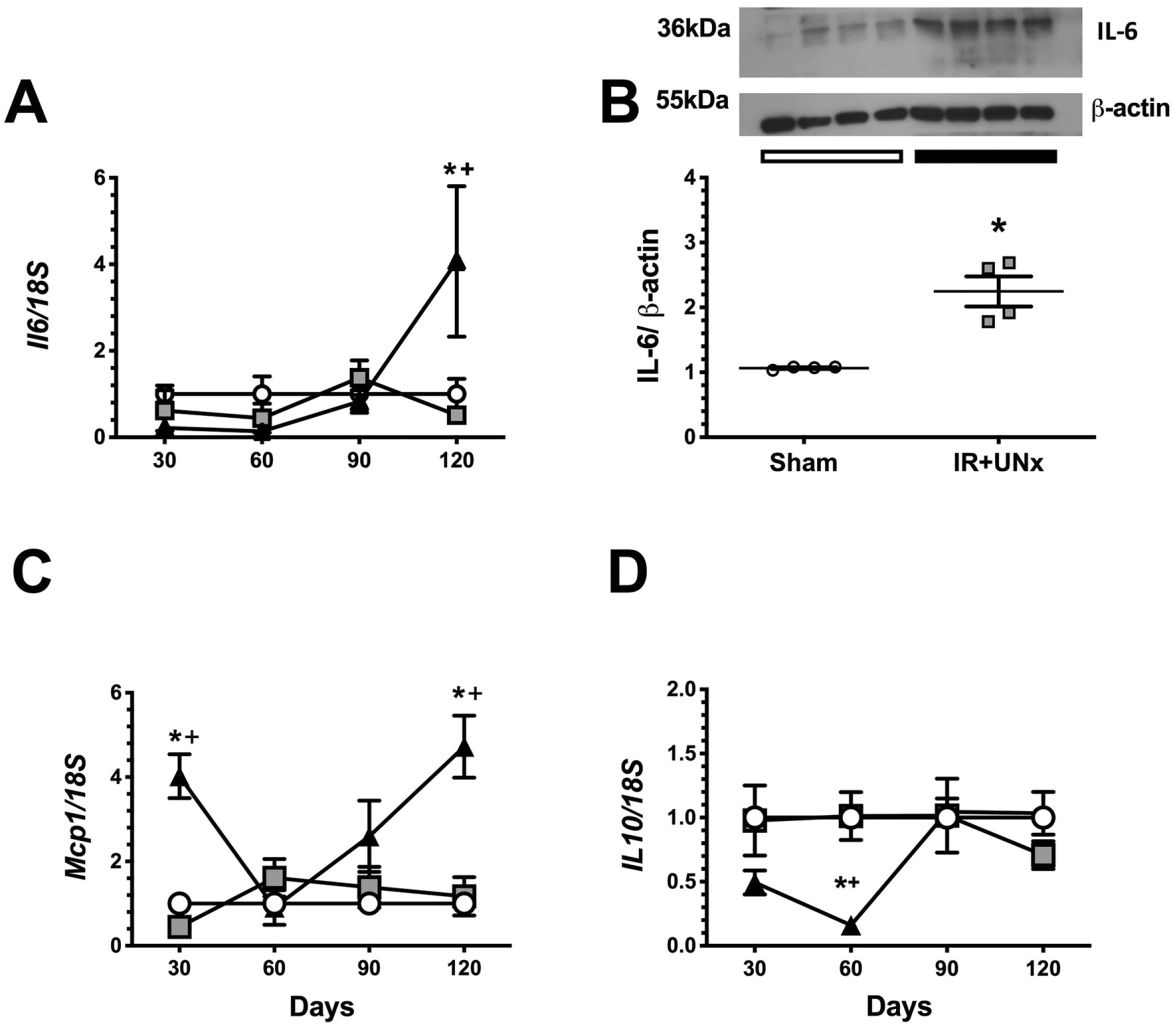
Inflammatory mediators participation during AKI to CKD transition. (**A**) mRNA levels of *Il6*, (**B**) Protein levels of IL-6 at fourth-month post-ischemia, including a representative cropped blots, (**C**) mRNA levels of *Mcp1* and (**D**) mRNA levels of *Il10*. Data are represented as the mean ± SE. n = 4, 4, and 5 for the S, UNx, and IR + UNx groups in each studied period: 30, 60, 90, and 120 days post-ischemia. White circles/bar represent the S, gray squares represent the UNx and black triangles/bar represent the IR + UNx groups. The one-way analysis of variance (ANOVA) was used to determine statistical differences, using the Bonferroni correction for multiple comparisons. *p < 0.05 versus S group and + *p* < 0.05 versus UNx group in their respective period. Full-length blots are presented in Supplementary Fig. 7.

The mRNA levels of interleukin 6 (*Il6*), monocyte chemoattract protein (*Mcp1*), and interleukin 10 (*Il10*) were measured throughout the study. *Il6* was upregulated in the IR + UNx group in the fourth-month after renal ischemia, as demonstrated by the significant elevation in interleukin 6 mRNA and protein levels (Fig. 5A, B). Because, we only observed a significant increase in *Il6* mRNA levels in the IR + UNx group at fourth-month post-ischemia, the IL6 protein levels were only evaluated in the Sham and IR + UNx groups in this specific time of the study. *Mcp1* mRNA levels increased in the first-month, and this elevation was observed again in the fourth month (Fig. 5C). The mRNA levels of the anti-inflammatory cytokine *Il10* showed a reduction starting in the first-month that became significant in the second month and returned to normal levels by the third month compared to that of the S and UNx groups (Fig. 5D).

These findings suggest that oxidative stress, rather than renal inflammation, is an initial trigger of the subsequent damage.

### Hypoxic response in the timing of CKD progression

**Figure 6:**
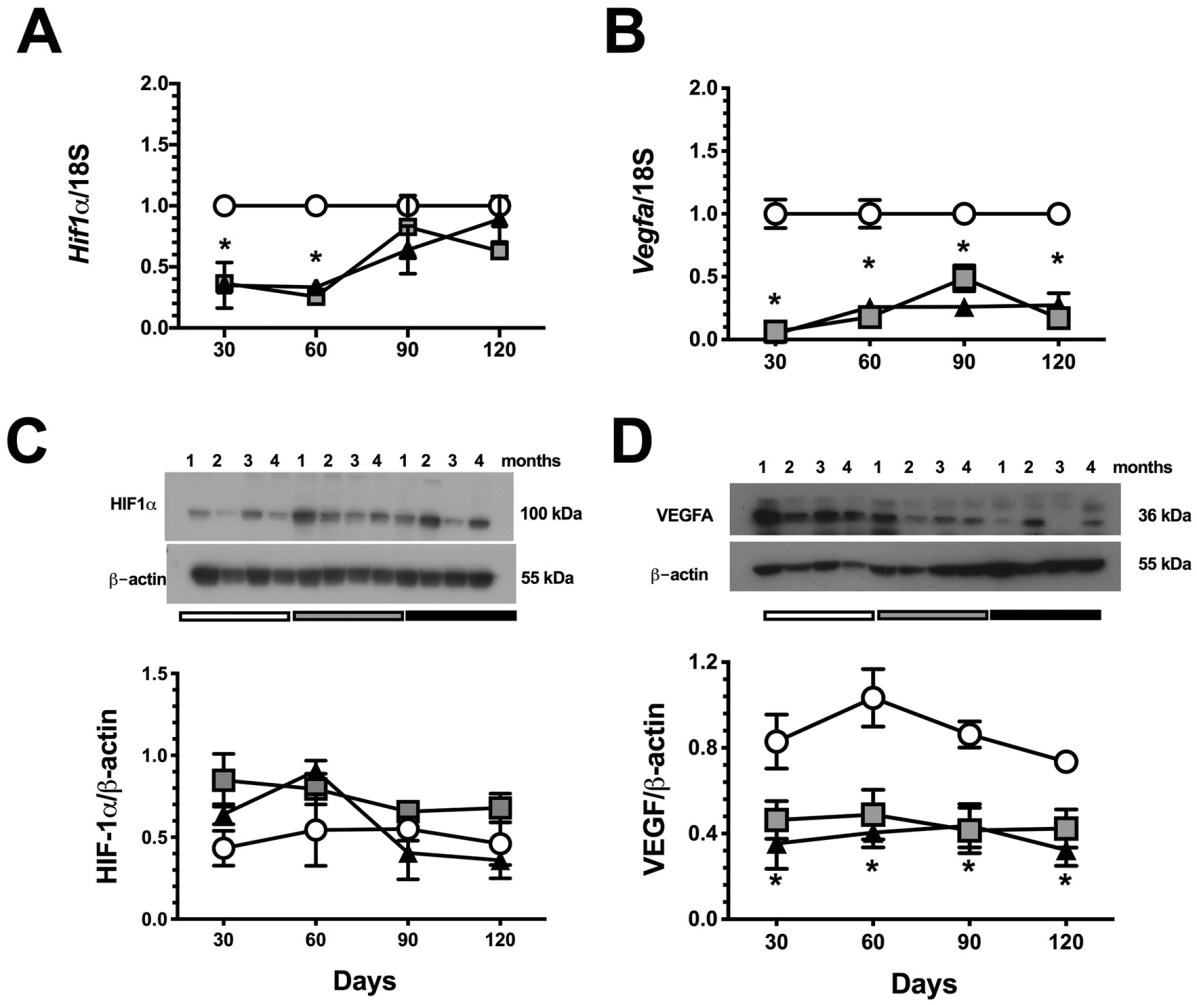
Temporal course of HIF-1α signaling during AKI to CKD transition. (**A**) mRNA levels of *Hif1a*, (**B**) mRNA levels of *Vegfa*, (**C**) Protein levels of HIF-1α, including a representative cropped blot, and (**D**) Protein levels of VEGF, including a representative cropped blot. Data are represented as the mean ± SE. n = 4, for S, UNx, and IR + UNx groups, in each studied period: 30, 60, 90, and 120 days post-ischemia. White circles represent the S, gray squares represent the UNx and black triangles represent the IR + UNx groups. The one-way analysis of variance (ANOVA) was used to determine statistical differences, using the Bonferroni correction for multiple comparisons. *p < 0.05 versus S group and + *p* < 0.05 versus UNx group in their respective period. Full-length blots are presented in Supplementary Figs. 8 and 9.

As we showed, the UNx group exhibited renal hyperperfusion starting in the first-month post ischemia (Fig. 2E). In contrast, because renal compensatory hyperperfusion was absent in the IR + UNx group, the HIF1α signaling pathway was studied. The gene expression *Hif1a* and one of its target genes, *Vegfa*, were assessed during AKI to CKD transition. In the UNx group, *Hif1a* mRNA levels were significantly reduced in the first and second-month post-ischemia, whereas HIF1α protein levels remained unaltered during follow-up. This response was explained and expected in part by the compensatory renal hyperperfusion seen in these rats. In contrast, there was an inefficient response to hypoxia in the IR + UNx group that exhibited renal hypoperfusion because there was a significant decrease in *Hif1a* mRNA levels in the first and second-month after ischemia (Fig. 6A). In support of this inefficient response, *Vegfa* mRNA and protein levels were significantly reduced starting in the first-month and remained so on during follow-up (Fig. 6B–D), despite *Hif1a* mRNA and protein levels being reestablished by the third-month, suggesting that an independent mechanism maintains *Vegfa* gene expression downregulation and could be related with the vascular rarefaction characteristic of the AKI to CKD transition^29–31^.

### Time course global DNA methylation during CKD progression induced by AKI

**Figure 7:**
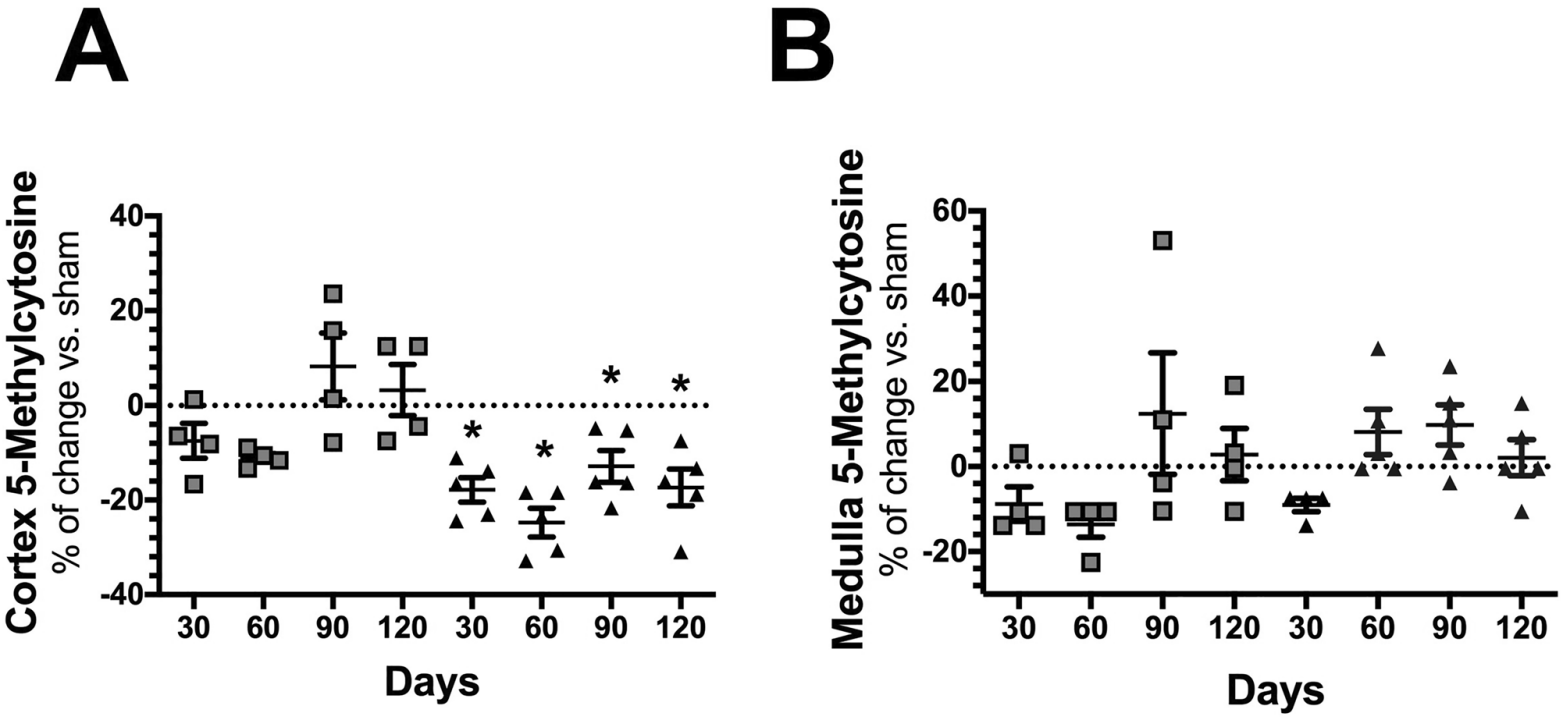
Global DNA methylation over the course of AKI to CKD transition. (**A**) Percentage change of global DNA methylation in renal cortex from the UNx (gray squares) and IR + UNx (black triangles) groups. (**B**) Percentage change of global DNA methylation in renal medulla from the UNx (gray squares) and IR + UNx (black triangles) groups, for each studied period: 30, 60, 90, and 120 days The one-way analysis of variance (ANOVA) was used to determine statistical differences, using the Bonferroni correction for multiple comparisons. *p < 0.05 versus S group.

Numerous studies have demonstrated that renal injury is associated with epigenetic changes, including histone modifications, DNA methylation, and the expression of various non‐coding RNAs^43^. We found that rats experiencing the AKI to CKD transition exhibited mainly hypomethylation of global DNA, which started in the first-month post-ischemia and was maintained throughout follow-up (Fig. 7A). These changes were only seen in the renal cortex, whereas no differences were observed in the renal medulla (Fig. 7B). In general, the renal medulla exhibited lower levels of DNA methylation compared to that of the renal cortex.

Based on the changes observed in the global methylation of DNA and the possible independent mechanisms regulating the decreased expression of VEGF in the AKI to CKD transition, we decided to evaluate the specific methylation of the *Vegfa* gene promoter**.**

### Vegfa gene promoter DNA methylation during AKI to CKD transition

**Figure 8:**
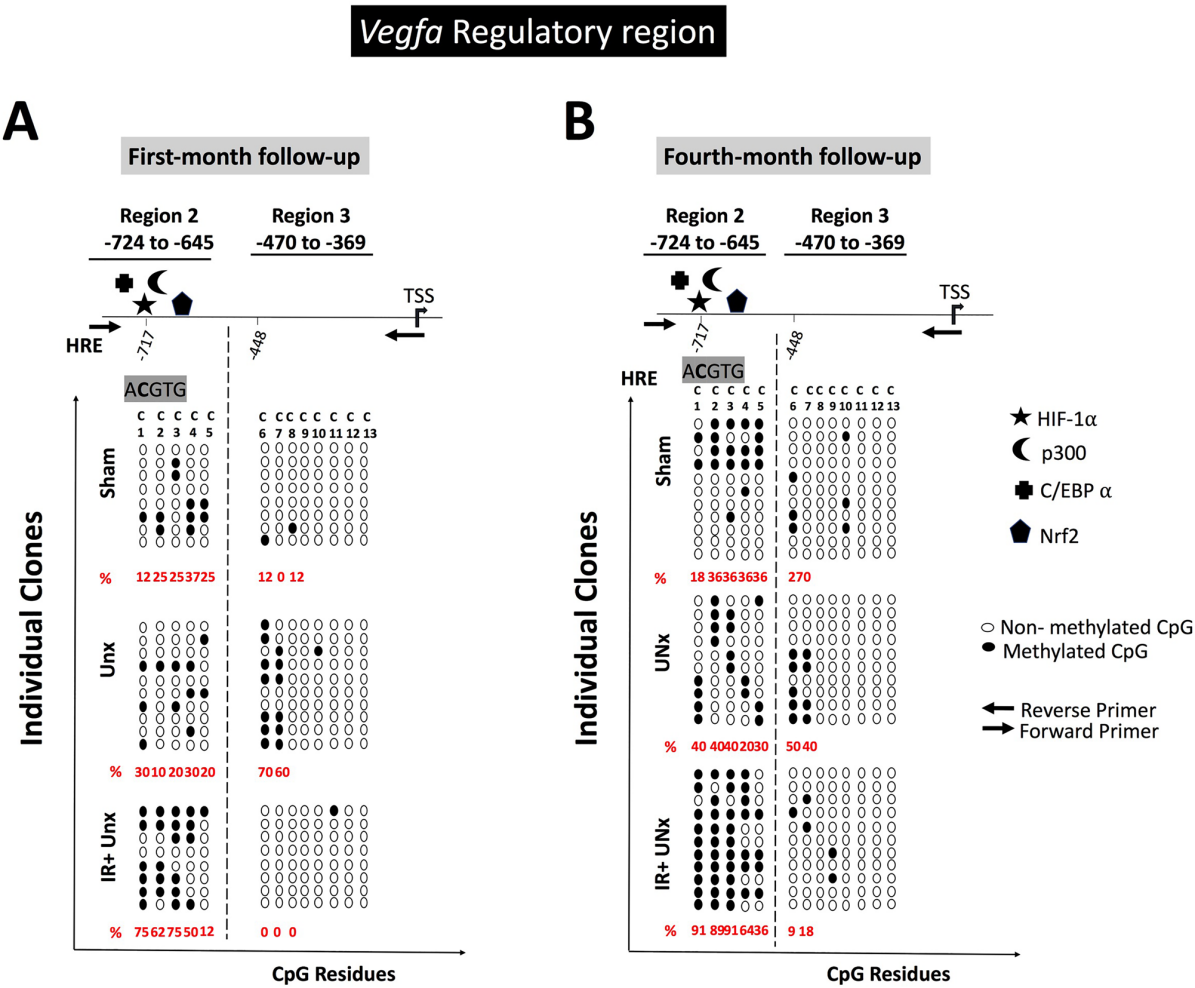
Bisulfite sequencing of two non-codifying upstream regions of *Vegfa* promoter for (**A**) the first-month and (**B**) the fourth month of follow-up for the Sham, UNx and IR + UNx groups, respectively. Arrows represented the forward and reverse primers for each amplicon. HIF-1α, p300, C/EBPα, and Nrf2 binding sites as is stated. White circles represented non-methylated CpG, and black circles represented methylated CpG, each circle represented an individual clone. Each C represented a cytosine in CpG context. TSS-Transcription star site; HRE-Hypoxia response element (ACGTG).

With the interest to know if DNA methylation was associated with the modulation of *Vegfa* and *Hif1a* expression in AKI to CKD transition, we assessed the methylation state on 5′-upstream promoter region by bisulfite sequencing. According to the decreased *Vegfa* gene expression, we localized a DNA hypermethylated region in the noncoding upstream region of this gene, since the first-month post-ischemia (Fig. 8A), which was maintained until the fourth-month of follow-up (Fig. 8B). Therefore, we discovered that in the binding site for HIF1α, located at the region 2 of the *Vegfa* gene promoter, contains a specific CpG that was highly methylated in the IR + UNx group since the first-month and (75%) compared to that of the S (12%) and UNx groups (30%) (Fig. 8A). Moreover, the hypermethylated region was maintained at the end of the study in the IR + UNx group (91%) compared to that of the S (18%) and UNx (40%) groups (Fig. 8B).

Consistent with our transcriptional findings, we did not found any methylation in the noncoding upstream promoter region of *Hif1a* among the groups (Suppl. Fig. 2).

## Discussion

Acute kidney injury is a public health problem and despite the advances in modern medicine, its incidence has not diminished in recent decades^44^. Also, the alarming increase in patients with CKD worldwide^45^, coupled with the recent recognition that AKI is an independent risk factor for CKD development^1,2^ requires joint work between biomedical and clinical researchers to avoid this complication. Therefore, it is imperative to study the temporality of the mechanisms that affect and lead to the AKI to CKD transition, which will allow for the identification of the key points and mediators in the development of this disease and propose specific therapeutic targets according to the stages of CKD. Many efforts have been made, and some pathways, such as trans-differentiation of pericytes into myofibroblasts^19,23^, uncontrolled proliferation of epithelial cells^22^, excessive production of TGFβ by both tubular epithelium^22^ and local myofibroblasts^25,26^, accumulation of extracellular matrix proteins^27,28^, chronic hypoxia^30,32^, vascular rarefaction^29,31,46^, and chronic stress of the endoplasmic reticulum^33^, have been identified, but many others remain to be elucidated.

In this study, we evaluated ischemic renal damage after 24 h of reperfusion plus right nephrectomy compared to that of the S and UNx groups. AKI induced by IR was characterized by elevated proteinuria, renal dysfunction, and oxidative stress. All IR + UNx rats exhibited the same magnitude of AKI. In the long term, the nephrectomy plus I/R model was able to accelerate the development of CKD. After four months, the IR + UNx group presented a progressive increase in proteinuria and a significant decrease in renal function. Among the alterations that occurred in the early phase of the transition, we found a significant increase in oxidative stress that was maintained throughout the study follow-up and a significant decrease in global DNA methylation, suggesting that both are early key players in the AKI to CKD transition.

As expected, the UNx group exhibited renal hyperperfusion and hyperfiltration to compensate the renal mass reduction for maintaining renal function within standard values, as the S group had. Interestingly, the IR + UNx group displayed sustained hypoperfusion that was observed beginning at an early phase and impacted the expected renal function compensation. Our results suggest that this poor renal hemodynamic response after an AKI episode could be one of the responsible mechanisms involved in the AKI to CKD transition, contributing to chronic renal hypoxia.

Previous studies have demonstrated that chronic hypoxia is a trigger mechanism in the AKI to CKD transition that coordinates the interaction between inflammation, oxidative stress, and progressive fibrosis^13,16–18,20–22,47^. Hif1α serves as a master regulator of adaptive responses against hypoxia, although, the levels of HIF1α can also be regulated by oxygen independent pathways^48^. This transcription factor induces an angiogenic pathway throughout the induction of *Vegfa* gene expression^49,50^. In this regard, we found an inefficient response against renal hypoxia in the IR + UNx group. *Hif1α* mRNA levels were decreased in the first and second-month post-ischemia, similar to those observed in the UNx group. Although, this response could be explained in the UNx group due to the compensatory elevation in RBF, which when normalized by kidney weight was similar to the S group. Since, HIF1α is regulated by proteasomal degradation pathways in response to oxygen levels^51^, it is very likely that in the UNx group, there is a greater metabolic work to maintain renal function and therefore, the state of renal oxygenation could not have changed. But this HIF1α response was unexpected in the IR + UNx group that exhibited renal hypoperfusion, a condition in which proteasomal degradation is not expected to occur. This inefficient response of HIF1α was also demonstrated by the reduction in its target gene *Vegfa*. Thus, *Vegfa* mRNA levels were reduced starting in the first-month, and interestingly, they remained reduced throughout the study, despite the restoration of HIF1α levels. The decreased *Vegfa* expression was also corroborated at the protein level throughout the study. Recent studies have reported that the late response of HIF1α could be related to the activation of inflammatory processes and the generation of renal fibrosis^52^. It is well known that the reduction in *Vegfa* gene expression is partly responsible for vascular rarefaction^46,53–55^ that accompanies the AKI to CKD transition, which also perpetuates chronic hypoxia. Although *Hif1a* mRNA levels were restored at the end of the study and protein levels remained unaltered, *Vegfa* levels continued diminishing; this opened the possibility of an alternative regulation of *Vegfa* gene expression through the involvement of epigenetic mechanisms.

It has been shown that the hypoxia response element (HRE) in the *Vegfa* promoter gene contains several cytosines in a CpG context that are potentially methylated. This methylation could reduce the HIF1α interaction with the *Vegfa* gene promoter^56,57^. In vitro studies have demonstrated that DNA hypermethylation in the *Vegfa* promoter region induces silencing of this gene^58^. To understand the reduction in *Vegfa* expression despite sustained renal hypoperfusion and recovery of *Hif1a* mRNA levels, we analyzed the methylation of the noncoding upstream region of *Vegfa* during the AKI to CKD transition. Interestingly, we found DNA hypermethylation in the promoter region of *Vegfa*. More importantly, we demonstrated that the HRE, as well as the core region for HIF1α interaction was hypermethylated since the first-month post-ischemia and it was maintained at the fourth-month of the follow-up. This site matches with the region 2, previously described to be important in *Vegf* gene transcription regulation^59^. Using the transcription factor binding site predictor tool PROMO (version 8.3 of TRANSFAC, http://alggen.lsi.upc.es), TFsitescan (http://www.ifti.org/cgi-bin/ifti/Tfsitescan.pl) and JASPAR database (http://dbcat.cgm.ntu.edu.tw), we found that this hypermethylated region contains a putative binding sequence responsible for the interaction of Hif1α, C/EBPα and the p300. This complex is essential for *Vegfa* transcription regulation and contributes to the pro-angiogenic pathway^60,61^. Our findings suggest that the reduction in *Vegfa* gene expression in the IR + UNx group resulted from epigenetic regulation, which could be partially responsible for inducing vascular rarefaction and chronic renal hypoxia, which are mechanisms implicated in the AKI to CKD transition^19,29–31,46^. In agreement with our results, it has been demonstrated that treatment with VEGF-121 was effective in suppressing the AKI to CKD transition induced by IR in rats. Although VEGF-121 did not affect AKI, the loss of peritubular capillaries in the cortex and outer stripe of the outer medulla was significantly attenuated^55^. Further experimental and clinical studies are required to evaluate VEGF therapeutic power in preventing the AKI to CKD transition. In this hypermethylated region, we also found a putative binding site for Nrf2, which is a master regulator of the antioxidant response^62^. Because hypermethylation of the *Vegfa* promoter region occurred from the first-month post-ischemia, it suggests that this epigenetic mechanism plays an important role in the onset of the disease, promoting chronic hypoxia and concomitant development of renal fibrosis. In a recent study, it was observed that indeed, epigenetic modifications occur early after folic acid-induced kidney damage. In particular, it was observed that de novo methylation of histone H3K4 is necessary for the differentiated cells to re-enter mitosis and regenerate the proximal tubular epithelium^63^. These findings together, highlighted the crucial role of the early epigenetic modifications in the long consequences after an ischemic insult. Furthermore, our results open an exciting research field to explore the mechanisms by which hypermethylation of the *Vegfa* promoter gene is occurring, in which cell subpopulations occur, as well as the molecular mediators of this phenomenon, such as histone modifications and the enzymes involved in this pathophysiological condition. In this context, previous reports have demonstrated that the up-regulation of DNA methyltransferase 1 (Dnmt1), DNA methylation, and transcriptional silencing are linked to fibroblast activation and kidney fibrosis^37^.

In addition to the mentioned changes, the IR + UNx group exhibited activation of renal inflammation. Pro-inflammatory molecules, as indicated by *Mcp1* and *Il6* mRNA levels, increased, while the anti-inflammatory molecule, *Il10*, decreased beginning at the early phase of the transition of this disease. These changes could result from the global DNA hypomethylation observed in the IR + UNx, as previous studies have shown in AKI, diabetic nephropathy, and CKD models^34,37,38^.

In summary, our study shows that early renal hypoperfusion, inefficient hypoxia response, increased oxidative stress, and increased inflammation play an important role in the AKI to CKD transition. Specifically, the inefficient hypoxia response results from the inadequate hypermethylation of the *Vegfa* promoter gene at the site of HIF1α binding that occurs in the early stage post-ischemia.

## Methods

All the experimental procedures in the animals were conducted following the Guide for the Care and Use of Laboratory Animals and were approved by the animal research ethics committee of the *Instituto Nacional de Ciencias Médicas y Nutrición Salvador Zubirán* with the approval number NMM-1852. The study was carried out in compliance with the ARRIVE guidelines.

### Experimental model: right nephrectomy and contralateral ischemia

Fifty-nine male Wistar rats weighing between 300 and 320 g were included. Seven rats did not meet our inclusion and exclusion criteria, two were excluded due to bleeding during the nephrectomy surgical procedure and five due to postoperative death in the first 72 h as a consequence of renal ischemia–reperfusion injury. Therefore, a total of 52 rats were included and randomly divided into three groups: the sham surgery group (S, n = 16), the right nephrectomy group (UNx, n = 16), and the group with right nephrectomy and simultaneous left renal ischemia of 45 min in the left kidney (IR + UNx, n = 20). Based on our previous experience with the IR experimental model, we calculated the sample size for comparison of two means, using the creatinine elevation after 24 h post-ischemia. Although, we did not use a method to generate the randomization sequence, each block of 3 rats was randomly assigned to each of the studied groups: S, UNx or IR + UNx and so on. The study was not blinded because no pharmacological intervention was carried out.

The animals were anesthetized with sodium pentobarbital (30 mg/kg) and kept in a thermoregulated bed to perform the surgery at 37 °C. Under anesthesia, an abdominal incision was made to expose the two kidneys, first, the nephrectomy of the right kidney was performed, dissecting the peri-renal fat, as well as, separating the adrenal gland from the kidney with delicacy to avoid damaging it. For the IR + UNx group, in addition to the nephrectomy, a clip was placed in the left kidney for 45 min, to induce the ischemic process and the reperfusion was achieved when the clip was removed, using the recovery of the coloration of the kidney, as an indicator. The animals were bred and kept in our animal facility; on a 12/12 h light/dark cycles and permitted ad libitum access to food and water. Each studied group was followed for 1, 2, 3, and 4 months, respectively. At the end of each experimental period, the following parameters were assessed: mean arterial pressure (MAP), renal blood flow (RBF), creatinine clearance, glomerular diameter, tubule-interstitial fibrosis, Ki67 positive cells, urinary H_2_O_2_ excretion urinary biomarkers of renal injury, RNA and protein levels of antioxidant enzymes and anti-inflammatory cytokines, DNA methylation and promoter VEGF methylation.

### Renal functional studies

The animals were placed in metabolic cages every month, to collect urine for at least 18 h to determine urinary protein excretion and creatinine clearance. A blood sample from the retro-orbital plexus was also collected monthly. Urine collections were carried out at the same schedule in all animals, starting between 4 and 6 pm and ended 18 h later, to avoid diurnal variations. For the determination of serum and urine creatinine, the colorimetric method of picric acid was used and quantified at 510 nm in a spectrophotometer. To calculate the creatinine clearance, the formula of C = (U*V)/S was used, where U is the urinary creatinine multiplied by the urinary volume (V), and S corresponds to the serum creatinine. Urinary protein excretion was determined by the turbidimetric method of trichloroacetic acid (TCA) and quantified at 420 nm in a spectrophotometer.

By the end of each experimental period, the animals were anesthetized with sodium pentobarbital (30 mg/kg) and were placed in a thermoregulated pad. The trachea was cannulated with a PE-240 polyethylene tube and the femoral arteries were catheterized with a polyethylene tube PE-90. The mean arterial pressure was recorded by one of the catheters placed in the femoral artery, using a pressure transducer (Model p23 db, Gould, Puerto Rico). Subsequently, an abdominal incision was made to expose the left kidney, the renal artery was dissected and an ultrasound probe was placed to register the renal blood flow (1RB, Transonic, Ithaca, NY).

The right kidney from the S group was ligated and removed and the left kidney upper and lower pole for the UNx and the IR + UNx groups were excised to separate renal medulla and cortex, both sections were immediately frozen at − 70 °C for further molecular analysis. The left kidney was then perfused through the femoral artery catheter with 20 mL of saline and then fixed with 20 mL of 4% formaldehyde, and removed immediately after. The animals were euthanized with an intraperitoneal delivery of an overdose of pentobarbital (100 mg/kg) after 1, 2, 3, and 4 months of renal reperfusion.

### Light microscopy and immunohistochemistry analysis

After tissue fixation, the kidneys were dehydrated and embedded in paraffin. Renal slices of 4 μm were obtained and stained with Periodic Acid Schiff (PAS) or Sirius red. In the slices stained with PAS, ten microphotographs (Magnification 400x) were obtained from different renal cortex fields of each kidney and glomerular diameter was quantified in at least 40 glomeruli per rat using a Nixon camera incorporated to the microscope. In the slices stained with Sirius red, five to eight subcortical periglomerular fields per section were randomly selected in kidneys from the groups studied (Magnification 100x). Tubulo-interstitial fibrosis consisted of extracellular matrix expansion with collagen deposition together with distortion and collapse of the tubules; fibrosis was evidenced by red coloration in Sirus red-stained slides. The affected area was delimited and the percentage of tubulointerstitial fibrosis was calculated by dividing the fibrotic by the total area, excluding the glomerular area. Researchers were blind to the experimental group.

To evaluate tubular cell proliferation, conventional immunoperoxidase assays for Ki67 (anti-Ki67 antibody, Abcam Cat. No. ab66155) were performed. For signal detection, HRP/DAB Detection System (Bio SB, Santa Barbara CA, USA Cat. No. BSB 0001) was used, slides were counterstain with hematoxylin. The number of Ki-67-positive epithelial cells on each slide was counted in at least 10-subcortical fields (100 × magnification).

### Hydrogen peroxide urinary excretion

In the urine collected during the follow-up time, the determination of urine hydrogen peroxides as an oxidative stress marker was carried out, using a commercial kit (Amplex Red Hydrogen Peroxide/Peroxidase Assay, Roche, Cat. No. A22188) following the manufacturer's instructions. The determination is based on the presence of peroxidase which, when reacted with hydrogen peroxide, produces a red-fluorescent compound, which was quantified in a spectrophotometer at 560 nm and extrapolated with a standard curve.

### RNA extraction and quantitative PCR

The total RNA was isolated from the kidneys using the TRIzol method (Invitrogen, Carlsbad, CA, Cat. No. 15596026) and checked for integrity using 1% agarose gel electrophoresis. To avoid DNA contamination, total RNA samples were treated with DNase (DNase I; Invitrogen, Carlsbad, CA, Cat. No. 18068015). Reverse transcription (RT) was carried out with 1 µg of total RNA and 200 U of Moloney murine leukemia virus reverse transcriptase (Invitrogen). The mRNA levels of *Hif1a*, *Vegfa*, pre-pro-endothelin (*Edn1*), endothelin receptor A (*Ednra*), endothelial NOS 3 (*NOS3),* transforming growth factor *(Tgfb1)*, monocyte chemoattractant protein *1 (Mcp1),* nuclear factor erythroid 2 like 2 *(Nfe2l2),* NADPH oxidase *4 (Nox4),* collagen-1 *(Col1a1)* interleukin 6 *(Il6), and* interleukin 10 *(Il10)* were quantified by real-time PCR on an ABI Prism 7300 Sequence Detection System (TaqMan, ABI, Foster City, CA, Cat. No. 4331182). Primers and probes were ordered as a kit as follows: *Hif1a*, (Rn0057756_m1), *Vegfa* (Rn01511602_m1), *Edn1* (Rn00561129_m1), *Ednra* (Rn00561137_m1), *NOS3* (Rn004352204_m1), *Tgfb1* (Rn00572010_m1), *Mcp1* (Rn00580555_m1), *Nfe2l2* (Rn00582415_m1), *Nox4* (Rn00585380_m1), *Col1a1* (Rn1463848_m1)*, Il6* (Rn01410330_m1), and *Il10* (Rn99999012_m1). As an endogenous control, eukaryotic 18S rRNA (predesigned assay reagent Applied by ABI, external run, Rn03928990_g1, Cat. No. 4319413E was used. The relative quantification of each gene expression was performed with the comparative threshold cycle (Ct) method.

### Western blot and antibodies

The renal cortex proteins were homogenized with a lysis buffer containing: 50 mM HEPES pH 7.4, 250 mM NaCl, 5 mM EDTA, 0.1% NP-40 and complete protease inhibitor (Roche, Cat. No.. 11,697,498,001). The proteins concentration was assessed by Lowry protein assay (Bio-Rad, Cat. No. 5000113 and 5,000,114). Renal cortex protein levels were detected by Western blot, tissue proteins (20–40 µg) were electrophoresed in a denaturing 8.5% acrylamide gel with SDS. The samples were prepared with loading buffer in a 1:1 ratio with a final volume of 20 μL. The membranes were incubated with the primary antibody TGFβ (Thermo Fisher, Cat. No. MA5-15,065, 1:1000), HIF-1alpha (Abcam, Cat. No.. ab2185, 1:5000) VEGFA (Invitrogen, MA1-16,629, 1:5000), IL-6 (Abcam, Cat. No. ab9324, 1:1000), and HRP β-Actin antibody [AC-15] (Abcam, Cat. No. ab49900, 1:1,000,000) overnight at 4 °C. Three 10-min washes were performed with TBS-1 × Tween and then incubated with the secondary antibody coupled to HRP, anti-rabbit or anti-mouse IgG (Santa Cruz, Cat. No. sc-2031 or sc-2004, respectively 1: 5000). Tissue proteins assessed by Western blot were normalized by β-Actin detection.

### Detection of urinary biomarkers by western blot

Urinary HSP72 levels were detected by Western blot, each urine was diluted 1:10 in 0.9% saline solution, and 10 µL of each dilution was loaded and resolved by 8.5% SDS-PAGE, as previously described^39–42,64^. The membranes were incubated with mouse anti-HSP72 antibody (ENZO Life Sciences, Cat. No. ADI-SPA-819F, 1:5000 dilution) or KIM-1 (Boster, Cat. No. PA1632, 1:5000) overnight at 4 °C. Thereafter; membranes were incubated with a secondary antibody, HRP-conjugated goat anti-mouse IgG, or anti-rabbit IgG, respectively (1:5000, Cat. No. sc-2031 or sc-2004, respectively, Santa Cruz). The proteins were detected using a commercial chemiluminescence kit (Millipore, Cat. No. WBKLS0500) and were normalized by urinary creatinine (UCreat).

### Genomic DNA extraction and quantification of global DNA methylation

For genomic DNA extraction, 50 mg of tissue was homogenized in 200 μL of 1X PBS (10 mM PO4, 137 mM NaCl, and 2.7 mM KCl), 60 μL of digestion buffer (Tris–HCl 1 M, EDTA 0.5 M, SDS 10%, NaCl 5 M, pH = 8), then 26 μL of Proteinase K (Sigma, Cat. No. P2308-100 mg, 10 mg/mL) was added and kept on ice for 5 min. Subsequently, the mixture was left overnight at 56 °C, then the samples were treated with 3 μL of RNase A (Qiagen, Cat. No. 19101, 10 mg/mL) and incubated for 3 h at 37 °C. By the end, 250 μL of phenol–chloroform-isoamyl alcohol (Sigma, Cat. No. 77617-100ML) was added, centrifuged for 20 min, 16.1 g at 4 °C. Only the upper phase was taken and 83 μL of ammonium acetate (7.5 M) and 250 μL of absolute ethanol were added. The samples were incubated at − 20 °C overnight, centrifuged and the supernatant was discarded. Two washes were made with 250 μL of 70% ethanol. The pellet was re-suspended in 200 μL of DNase⁄RNase-Free Distilled Water. To analyze the global DNA methylation, the commercial methylation kit of 5mC DNA (Zymo Research Cat. No. D5326) was used, 100 ng of DNA from each sample was used and it was taken to a volume of 100 μL with 5mC of coating buffer. The mix was incubated at 98 °C for 5 min and then left on ice for 10 min. Then it was added to the plate and incubated at 37 °C for 1 h and the excess was discarded and washed with 200 μL of 5mC Elisa Buffer. Thereafter, 200 μL of 5mC Elisa buffer was added to each well and incubated at 37 °C for 30 min. A mixture of 5-methyl cytosine primary antibody (1:2,000) and HRP-coupled secondary anti-rabbit antibody (1:1000) was added. Finally, 100 μL of HRP developer was added to each well, incubated 1 h, and measured in a spectrometer at 405–450 nm. The results were extrapolated with a standard curve and the correction was made for the percentage of cytosines and guanine dinucleotides (CpGs), which have been previously reported for the rat genome^65^.

### Sequence analysis and bisulfite primer design

Three binding Hif1α sequences in the promoter *Vegf* gene in the rat have been previously identified and were named as region 1 (− 976 to − 857), region 2 (− 724 to − 645), and region 3 (− 470 to − 369), respectively (Suppl. Fig. 1)^59^. DataBase of CpG islands and Analytical Tool (DBCAT) (http://dbcat.cgm.ntu.edu.tw) software-assisted us to identify the CpG islands in the promoter and the first part of the coding regions of *Vegf* gene. This analysis showed that only regions 2 and 3 were enriched of CpG islands, suggesting that these regions are susceptible to methylation. Consequently, optimal primers were designed in the Methyl Primer Express software for bisulfite sequencing of *Hif-1α* and *Vegf* gene promoters. For *Hif-1α* the primers were: 5′-GTAGAGAGTAGAGATTGAGTT-3′ (forward) and 5′-CAAAACCTAACCAAACACTAC-3′ (reverse) that amplified the region from − 1390 to − 688 (702 bp): As was commented before there are three HIF1α binding sequences in the promoter of *Vegfa*, but only two are susceptible to methylation: the region 2, from − 724 to − 645, and the region 3. from − 470 to − 369, we amplified together with the following primers: 5′-GGTTTTGTTAGATTTTATAGTG-3′ (forward) and 5′-CCATAACCTAAAAATTATCTATC-3′ (reverse) yielding a product of 763 bp.

### Sodium bisulfite DNA conversion and sequencing

Genomic DNA (3 μg) was processed with sodium bisulfite^66^, DNA fragments of interest were PCR-amplified. The amplified DNA fragments were cloned into the pGEM-T Easy system (Promega, Cat. No. A1360), and Sanger sequenced using its respective reverse primer. At least 8 clones were evaluated for each region.

### Statistical analysis

The results are presented as the mean ± SE. The significance of the differences between groups was assessed by 1-way ANOVA using the Bonferroni correction for multiple comparisons. All comparisons passed the normality test. Statistical significance was defined when the *p*-value was < 0.05. All the graphs and statistical analyses were performed using the statistical GraphPad Prisma 8 software for Mac (GraphPad Software, San Diego, CA, USA).

## Supplementary Information

Supplementary Information 1.
