# Supplementary material for: Vegfa promoter gene hypermethylation at HIF1α binding site is an early contributor to CKD progression after renal ischemia
Source: Sci Rep. 2021 Apr 22;11:8769. doi: 10.1038/s41598-021-88000-5 (PMC8062449; doi:10.1038/s41598-021-88000-5)
Supplement: Supplementary file 1 — Supplementary Information 1. [file 41598_2021_88000_MOESM1_ESM.pptx]

## Slide 1
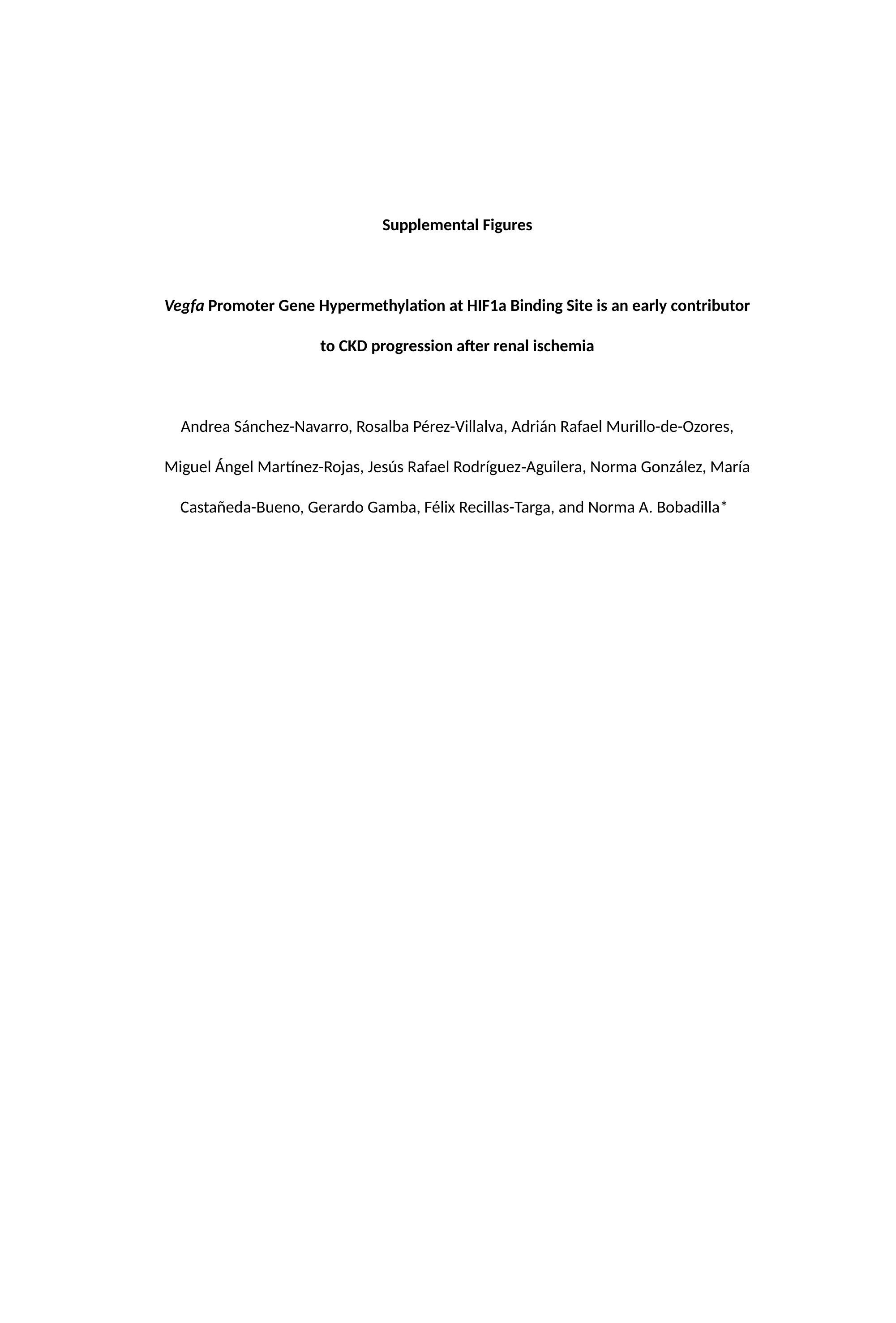

Supplemental Figures
Vegfa Promoter Gene Hypermethylation at HIF1a Binding Site is an early contributor to CKD progression after renal ischemia
Andrea Sánchez-Navarro, Rosalba Pérez-Villalva, Adrián Rafael Murillo-de-Ozores, Miguel Ángel Martínez-Rojas, Jesús Rafael Rodríguez‐Aguilera, Norma González, María Castañeda-Bueno, Gerardo Gamba, Félix Recillas-Targa, and Norma A. Bobadilla*

## Slide 2
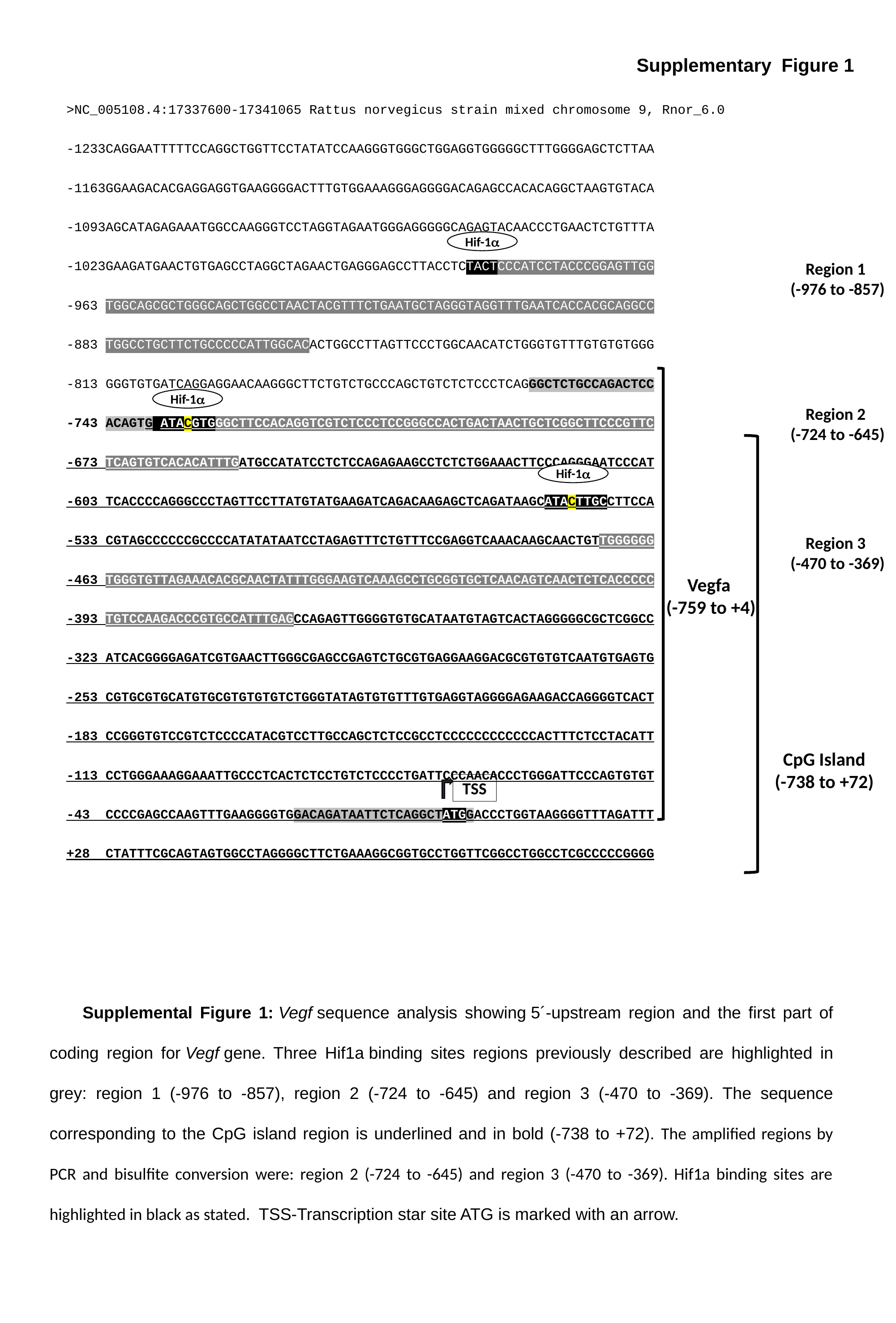

Supplementary Figure 1
>NC_005108.4:17337600-17341065 Rattus norvegicus strain mixed chromosome 9, Rnor_6.0
-1233CAGGAATTTTTCCAGGCTGGTTCCTATATCCAAGGGTGGGCTGGAGGTGGGGGCTTTGGGGAGCTCTTAA
-1163GGAAGACACGAGGAGGTGAAGGGGACTTTGTGGAAAGGGAGGGGACAGAGCCACACAGGCTAAGTGTACA
-1093AGCATAGAGAAATGGCCAAGGGTCCTAGGTAGAATGGGAGGGGGCAGAGTACAACCCTGAACTCTGTTTA
-1023GAAGATGAACTGTGAGCCTAGGCTAGAACTGAGGGAGCCTTACCTCTACTCCCATCCTACCCGGAGTTGG
-963 TGGCAGCGCTGGGCAGCTGGCCTAACTACGTTTCTGAATGCTAGGGTAGGTTTGAATCACCACGCAGGCC
-883 TGGCCTGCTTCTGCCCCCATTGGCACACTGGCCTTAGTTCCCTGGCAACATCTGGGTGTTTGTGTGTGGG
-813 GGGTGTGATCAGGAGGAACAAGGGCTTCTGTCTGCCCAGCTGTCTCTCCCTCAGGGCTCTGCCAGACTCC
-743 ACAGTGCATACGTGGGCTTCCACAGGTCGTCTCCCTCCGGGCCACTGACTAACTGCTCGGCTTCCCGTTC
-673 TCAGTGTCACACATTTGATGCCATATCCTCTCCAGAGAAGCCTCTCTGGAAACTTCCCAGGGAATCCCAT
-603 TCACCCCAGGGCCCTAGTTCCTTATGTATGAAGATCAGACAAGAGCTCAGATAAGCATACTTGCCTTCCA
-533 CGTAGCCCCCCGCCCCATATATAATCCTAGAGTTTCTGTTTCCGAGGTCAAACAAGCAACTGTTGGGGGG
-463 TGGGTGTTAGAAACACGCAACTATTTGGGAAGTCAAAGCCTGCGGTGCTCAACAGTCAACTCTCACCCCC
-393 TGTCCAAGACCCGTGCCATTTGAGCCAGAGTTGGGGTGTGCATAATGTAGTCACTAGGGGGCGCTCGGCC
-323 ATCACGGGGAGATCGTGAACTTGGGCGAGCCGAGTCTGCGTGAGGAAGGACGCGTGTGTCAATGTGAGTG
-253 CGTGCGTGCATGTGCGTGTGTGTCTGGGTATAGTGTGTTTGTGAGGTAGGGGAGAAGACCAGGGGTCACT
-183 CCGGGTGTCCGTCTCCCCATACGTCCTTGCCAGCTCTCCGCCTCCCCCCCCCCCCACTTTCTCCTACATT
-113 CCTGGGAAAGGAAATTGCCCTCACTCTCCTGTCTCCCCTGATTCCCAACACCCTGGGATTCCCAGTGTGT
-43 CCCCGAGCCAAGTTTGAAGGGGTGGACAGATAATTCTCAGGCTATGGACCCTGGTAAGGGGTTTAGATTT
+28 CTATTTCGCAGTAGTGGCCTAGGGGCTTCTGAAAGGCGGTGCCTGGTTCGGCCTGGCCTCGCCCCCGGGG
Hif-1a
Region 1
(-976 to -857)
Hif-1a
Region 2
(-724 to -645)
Hif-1a
Region 3
(-470 to -369)
Vegfa
(-759 to +4)
CpG Island
(-738 to +72)
TSS
Supplemental Figure 1: Vegf sequence analysis showing 5´-upstream region and the first part of coding region for Vegf gene. Three Hif1a binding sites regions previously described are highlighted in grey: region 1 (-976 to -857), region 2 (-724 to -645) and region 3 (-470 to -369). The sequence corresponding to the CpG island region is underlined and in bold (-738 to +72). The amplified regions by PCR and bisulfite conversion were: region 2 (-724 to -645) and region 3 (-470 to -369). Hif1a binding sites are highlighted in black as stated.  TSS-Transcription star site ATG is marked with an arrow.

## Slide 3
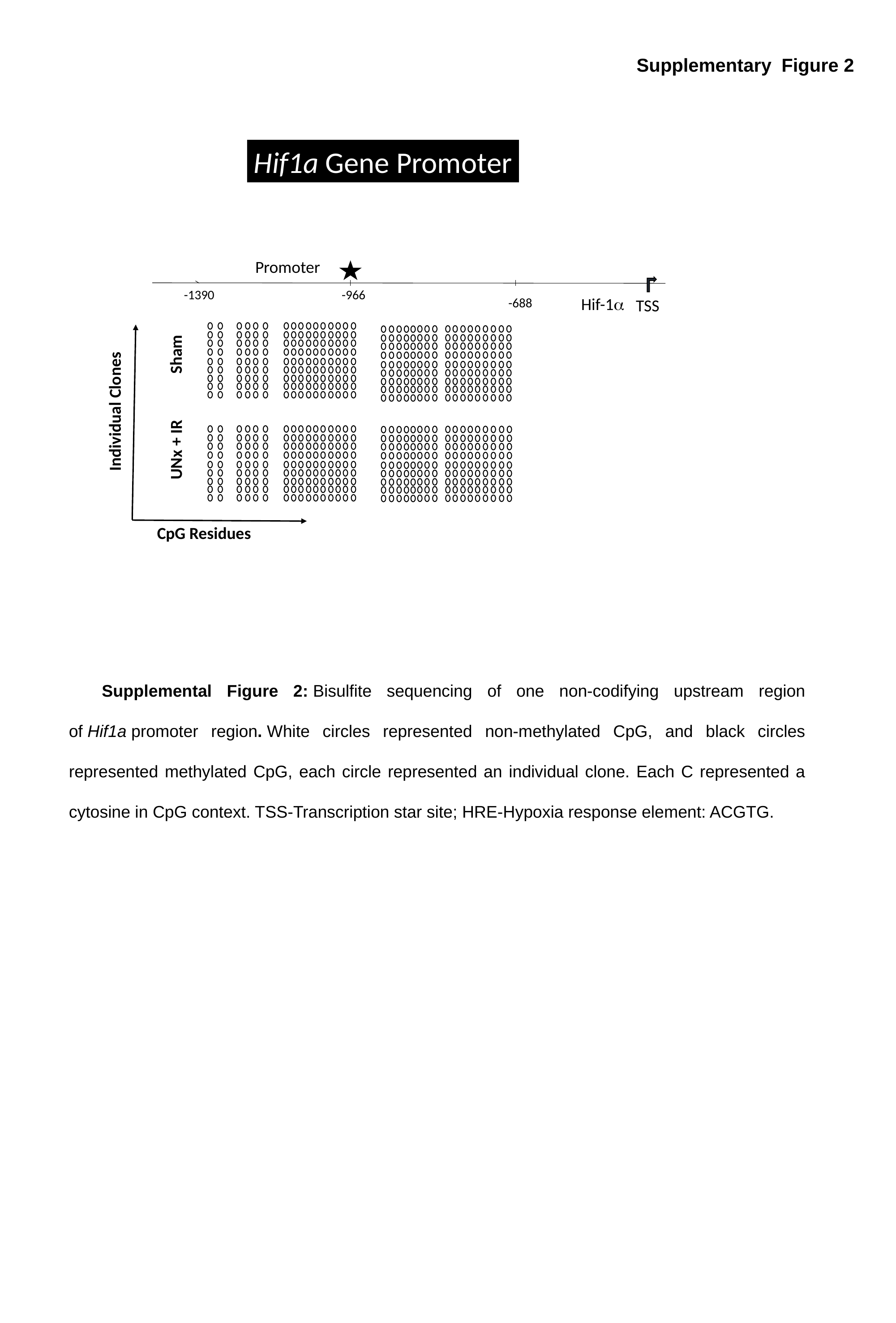

Supplementary Figure 2
Hif1a Gene Promoter
Promoter
-1390
-966
Hif-1a
-688
TSS
Individual Clones
CpG Residues
Sham
UNx + IR
Supplemental Figure 2: Bisulfite sequencing of one non-codifying upstream region of Hif1a promoter region. White circles represented non-methylated CpG, and black circles represented methylated CpG, each circle represented an individual clone. Each C represented a cytosine in CpG context. TSS-Transcription star site; HRE-Hypoxia response element: ACGTG.

## Slide 4
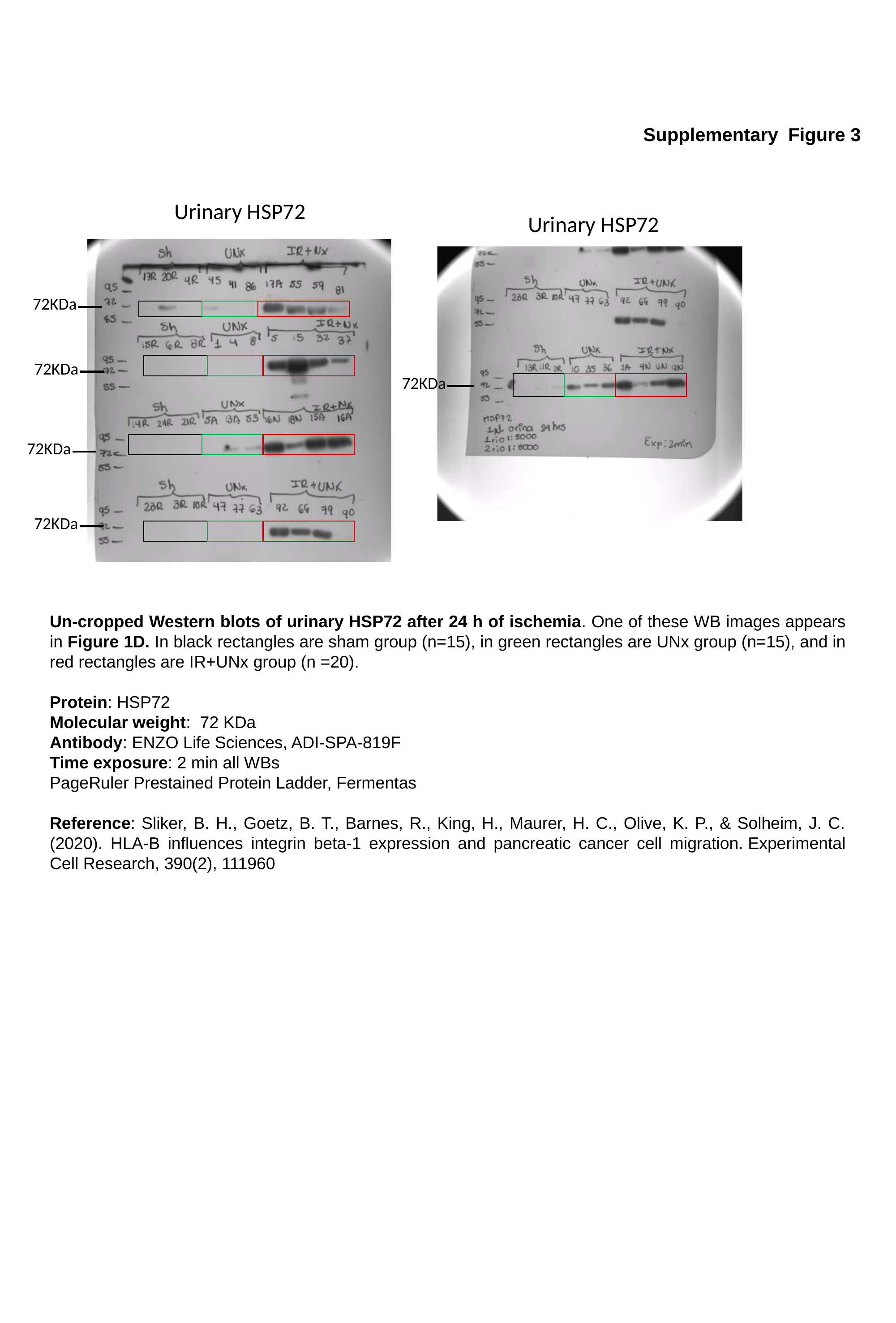

Supplementary Figure 3
Urinary HSP72
Urinary HSP72
72KDa
72KDa
72KDa
72KDa
72KDa
Un-cropped Western blots of urinary HSP72 after 24 h of ischemia. One of these WB images appears in Figure 1D. In black rectangles are sham group (n=15), in green rectangles are UNx group (n=15), and in red rectangles are IR+UNx group (n =20).
Protein: HSP72
Molecular weight: 72 KDa
Antibody: ENZO Life Sciences, ADI-SPA-819F
Time exposure: 2 min all WBs
PageRuler Prestained Protein Ladder, Fermentas
Reference: Sliker, B. H., Goetz, B. T., Barnes, R., King, H., Maurer, H. C., Olive, K. P., & Solheim, J. C. (2020). HLA-B influences integrin beta-1 expression and pancreatic cancer cell migration. Experimental Cell Research, 390(2), 111960

## Slide 5
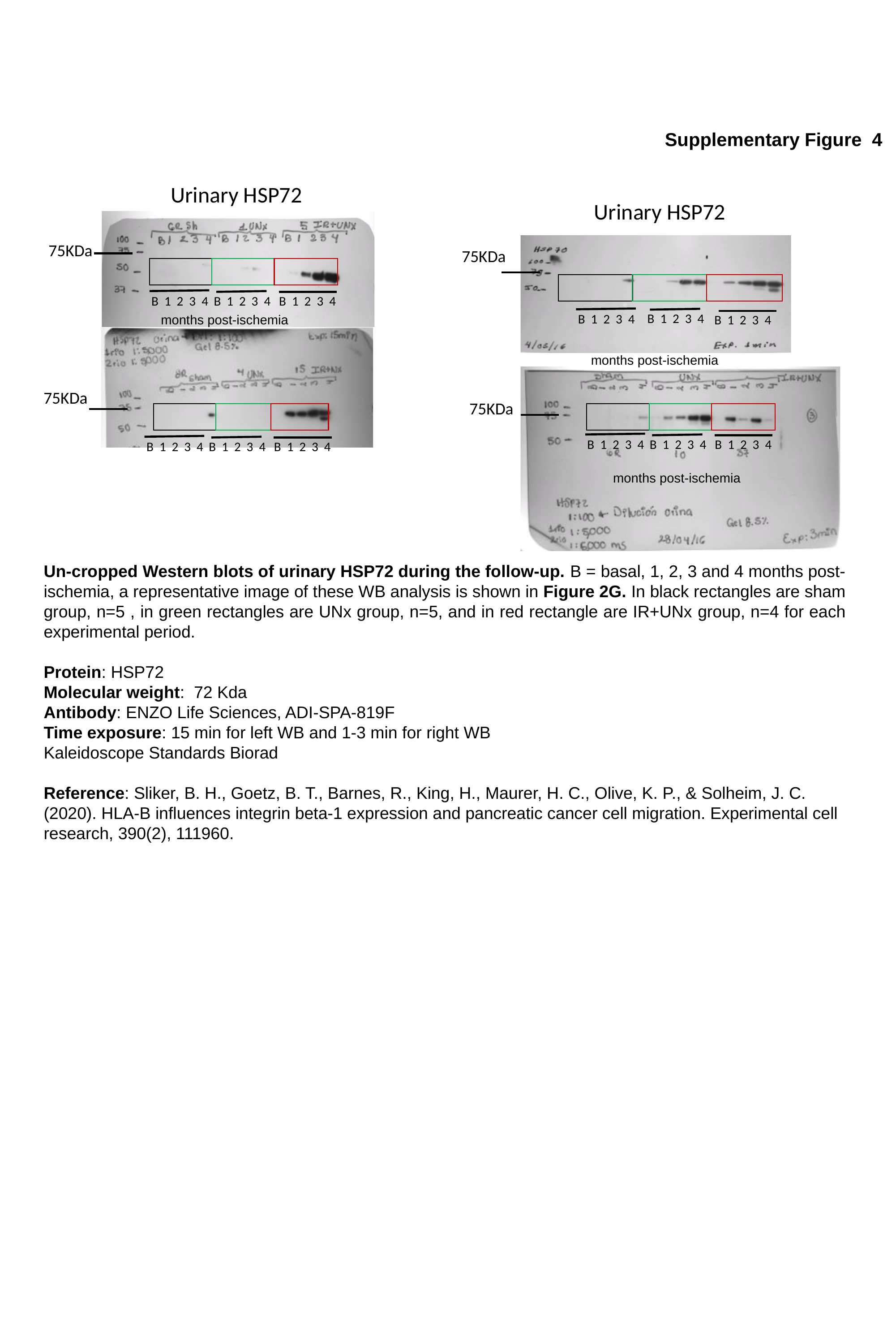

Supplementary Figure 4
Urinary HSP72
Urinary HSP72
75KDa
75KDa
B 1 2 3 4
B 1 2 3 4
B 1 2 3 4
B 1 2 3 4
B 1 2 3 4
months post-ischemia
B 1 2 3 4
months post-ischemia
75KDa
75KDa
B 1 2 3 4
B 1 2 3 4
B 1 2 3 4
B 1 2 3 4
B 1 2 3 4
B 1 2 3 4
months post-ischemia
Un-cropped Western blots of urinary HSP72 during the follow-up. B = basal, 1, 2, 3 and 4 months post-ischemia, a representative image of these WB analysis is shown in Figure 2G. In black rectangles are sham group, n=5 , in green rectangles are UNx group, n=5, and in red rectangle are IR+UNx group, n=4 for each experimental period.
Protein: HSP72
Molecular weight: 72 Kda
Antibody: ENZO Life Sciences, ADI-SPA-819F
Time exposure: 15 min for left WB and 1-3 min for right WB
Kaleidoscope Standards Biorad
Reference: Sliker, B. H., Goetz, B. T., Barnes, R., King, H., Maurer, H. C., Olive, K. P., & Solheim, J. C. (2020). HLA-B influences integrin beta-1 expression and pancreatic cancer cell migration. Experimental cell research, 390(2), 111960.

## Slide 6
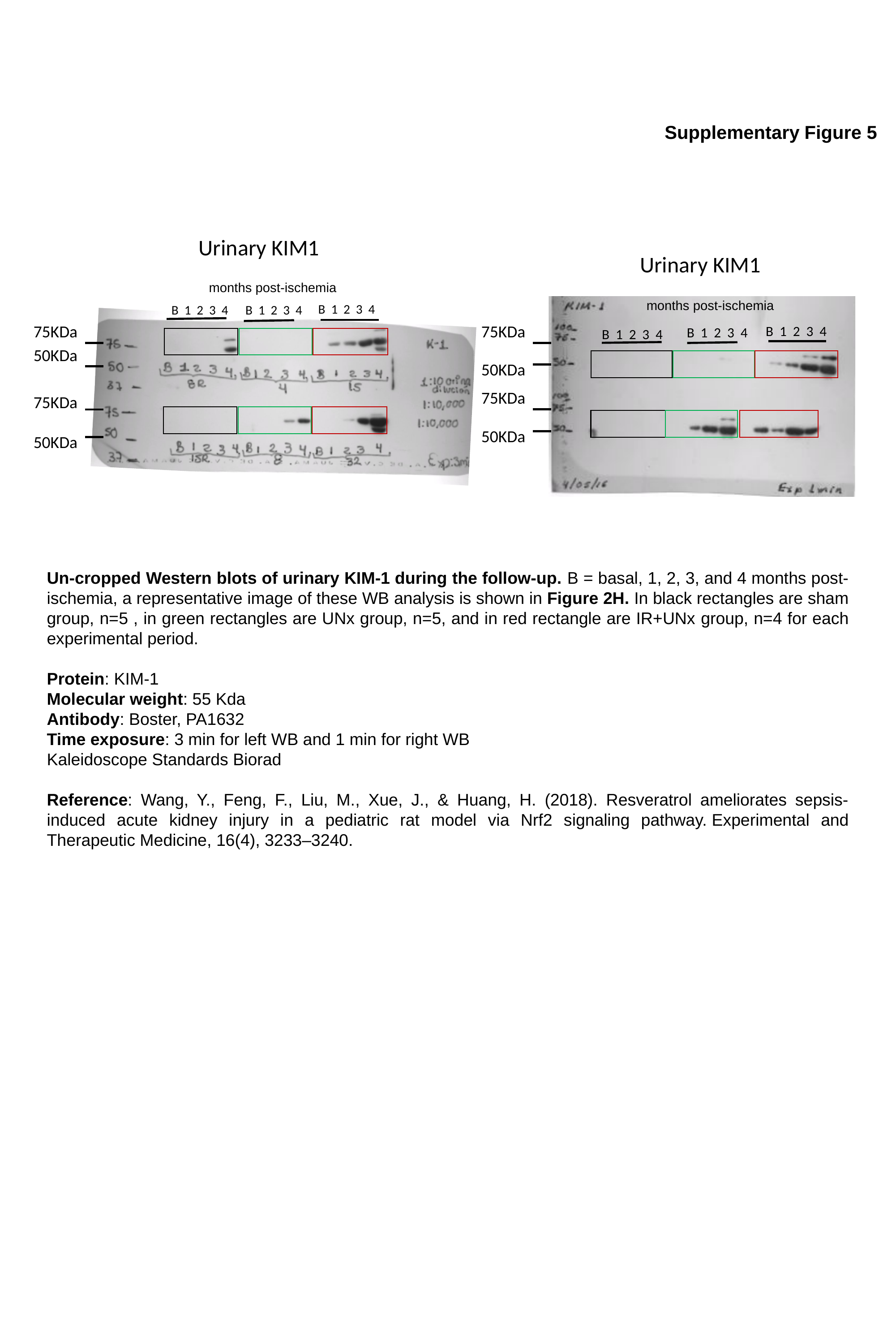

Supplementary Figure 5
Urinary KIM1
Urinary KIM1
months post-ischemia
months post-ischemia
B 1 2 3 4
B 1 2 3 4
B 1 2 3 4
75KDa
75KDa
B 1 2 3 4
B 1 2 3 4
B 1 2 3 4
50KDa
50KDa
75KDa
75KDa
50KDa
50KDa
Un-cropped Western blots of urinary KIM-1 during the follow-up. B = basal, 1, 2, 3, and 4 months post-ischemia, a representative image of these WB analysis is shown in Figure 2H. In black rectangles are sham group, n=5 , in green rectangles are UNx group, n=5, and in red rectangle are IR+UNx group, n=4 for each experimental period.
Protein: KIM-1
Molecular weight: 55 Kda
Antibody: Boster, PA1632
Time exposure: 3 min for left WB and 1 min for right WB
Kaleidoscope Standards Biorad
Reference: Wang, Y., Feng, F., Liu, M., Xue, J., & Huang, H. (2018). Resveratrol ameliorates sepsis-induced acute kidney injury in a pediatric rat model via Nrf2 signaling pathway. Experimental and Therapeutic Medicine, 16(4), 3233–3240.

## Slide 7
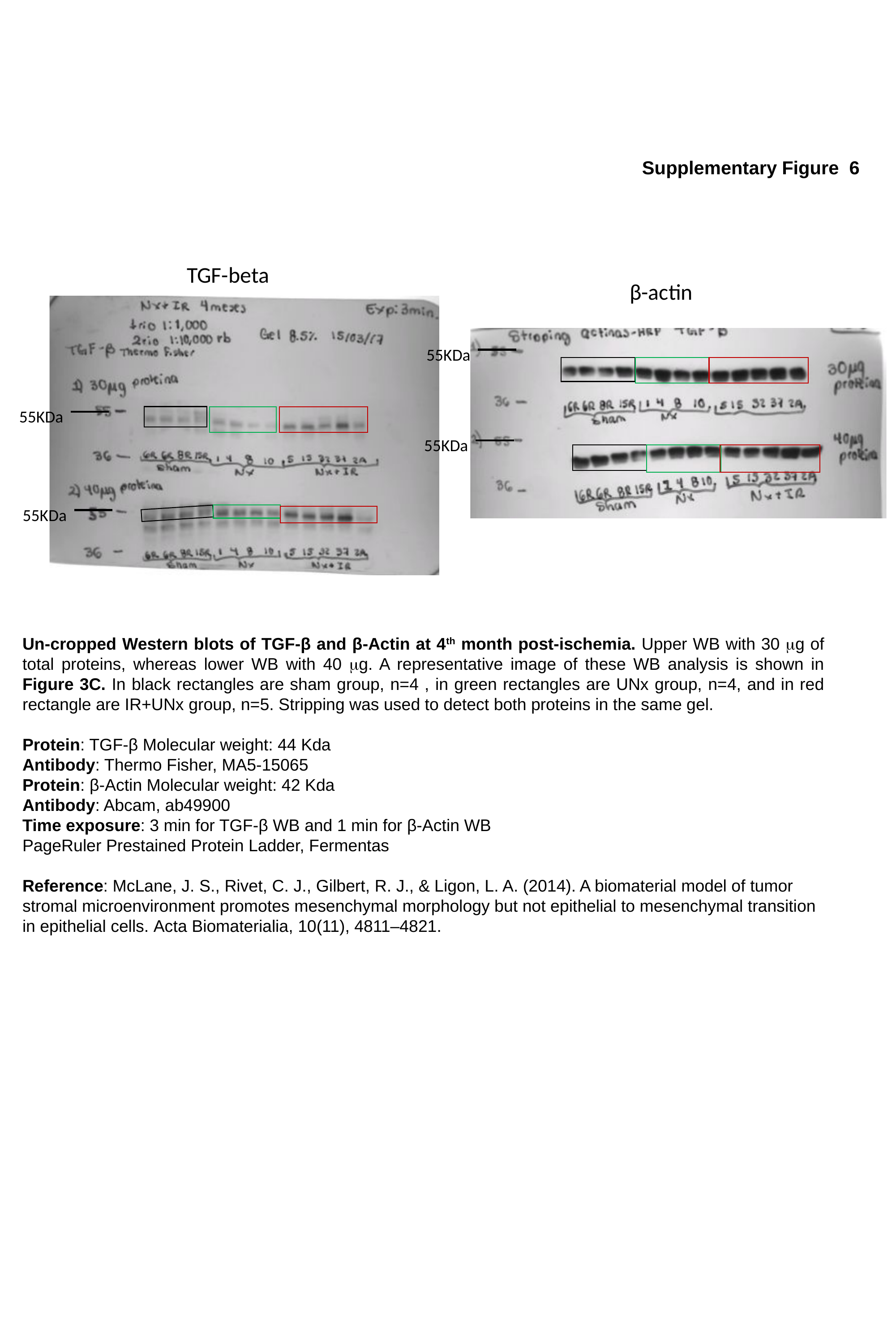

Supplementary Figure 6
TGF-beta
β-actin
55KDa
55KDa
55KDa
55KDa
Un-cropped Western blots of TGF-β and β-Actin at 4th month post-ischemia. Upper WB with 30 mg of total proteins, whereas lower WB with 40 mg. A representative image of these WB analysis is shown in Figure 3C. In black rectangles are sham group, n=4 , in green rectangles are UNx group, n=4, and in red rectangle are IR+UNx group, n=5. Stripping was used to detect both proteins in the same gel.
Protein: TGF-β Molecular weight: 44 Kda
Antibody: Thermo Fisher, MA5-15065
Protein: β-Actin Molecular weight: 42 Kda
Antibody: Abcam, ab49900
Time exposure: 3 min for TGF-β WB and 1 min for β-Actin WB
PageRuler Prestained Protein Ladder, Fermentas
Reference: McLane, J. S., Rivet, C. J., Gilbert, R. J., & Ligon, L. A. (2014). A biomaterial model of tumor stromal microenvironment promotes mesenchymal morphology but not epithelial to mesenchymal transition in epithelial cells. Acta Biomaterialia, 10(11), 4811–4821.
| |
| --- |

## Slide 8
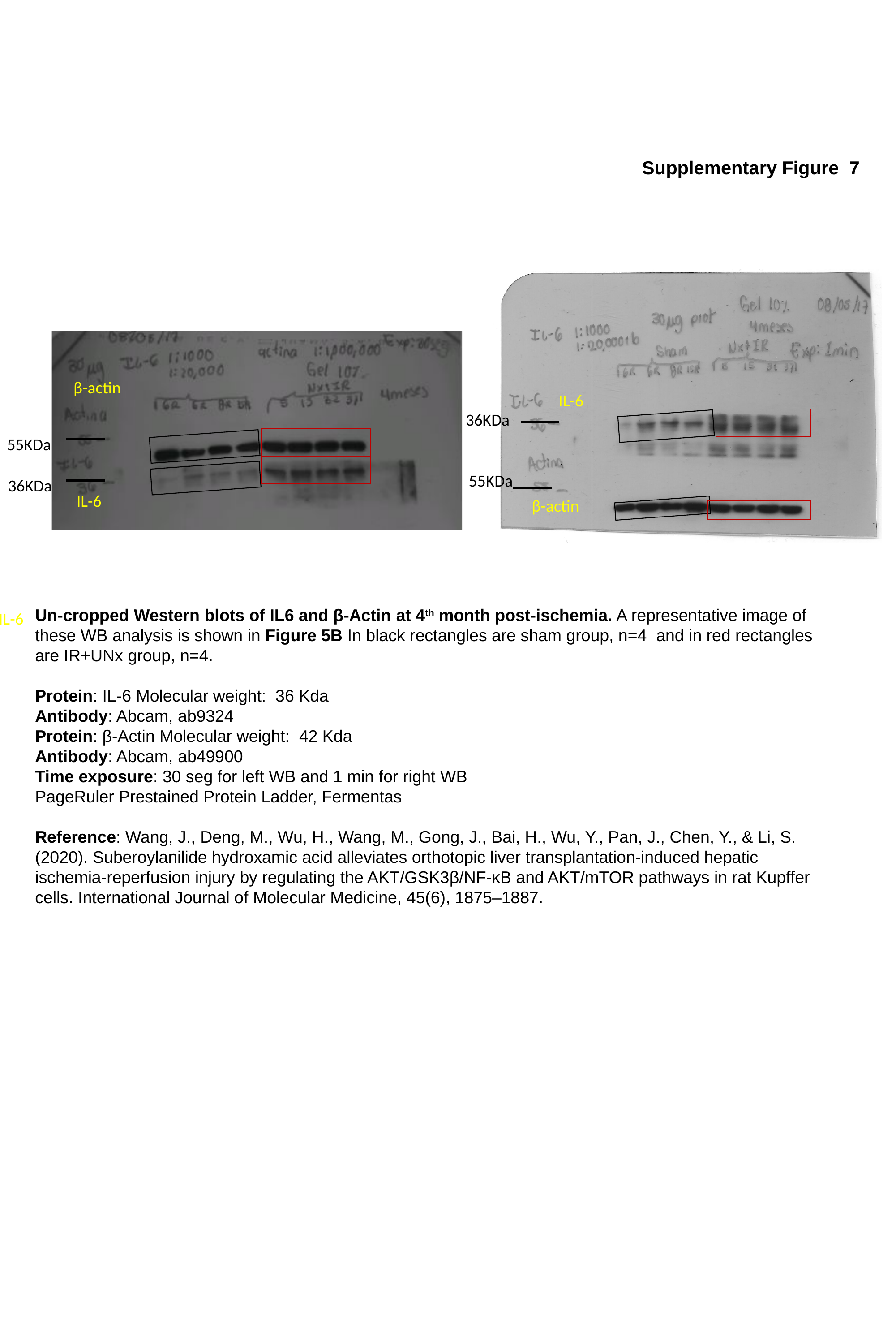

Supplementary Figure 7
β-actin
IL-6
36KDa
55KDa
55KDa
36KDa
IL-6
β-actin
Un-cropped Western blots of IL6 and β-Actin at 4th month post-ischemia. A representative image of these WB analysis is shown in Figure 5B In black rectangles are sham group, n=4 and in red rectangles are IR+UNx group, n=4.
Protein: IL-6 Molecular weight: 36 Kda
Antibody: Abcam, ab9324
Protein: β-Actin Molecular weight: 42 Kda
Antibody: Abcam, ab49900
Time exposure: 30 seg for left WB and 1 min for right WB
PageRuler Prestained Protein Ladder, Fermentas
Reference: Wang, J., Deng, M., Wu, H., Wang, M., Gong, J., Bai, H., Wu, Y., Pan, J., Chen, Y., & Li, S. (2020). Suberoylanilide hydroxamic acid alleviates orthotopic liver transplantation‑induced hepatic ischemia‑reperfusion injury by regulating the AKT/GSK3β/NF‑κB and AKT/mTOR pathways in rat Kupffer cells. International Journal of Molecular Medicine, 45(6), 1875–1887.
IL-6

## Slide 9
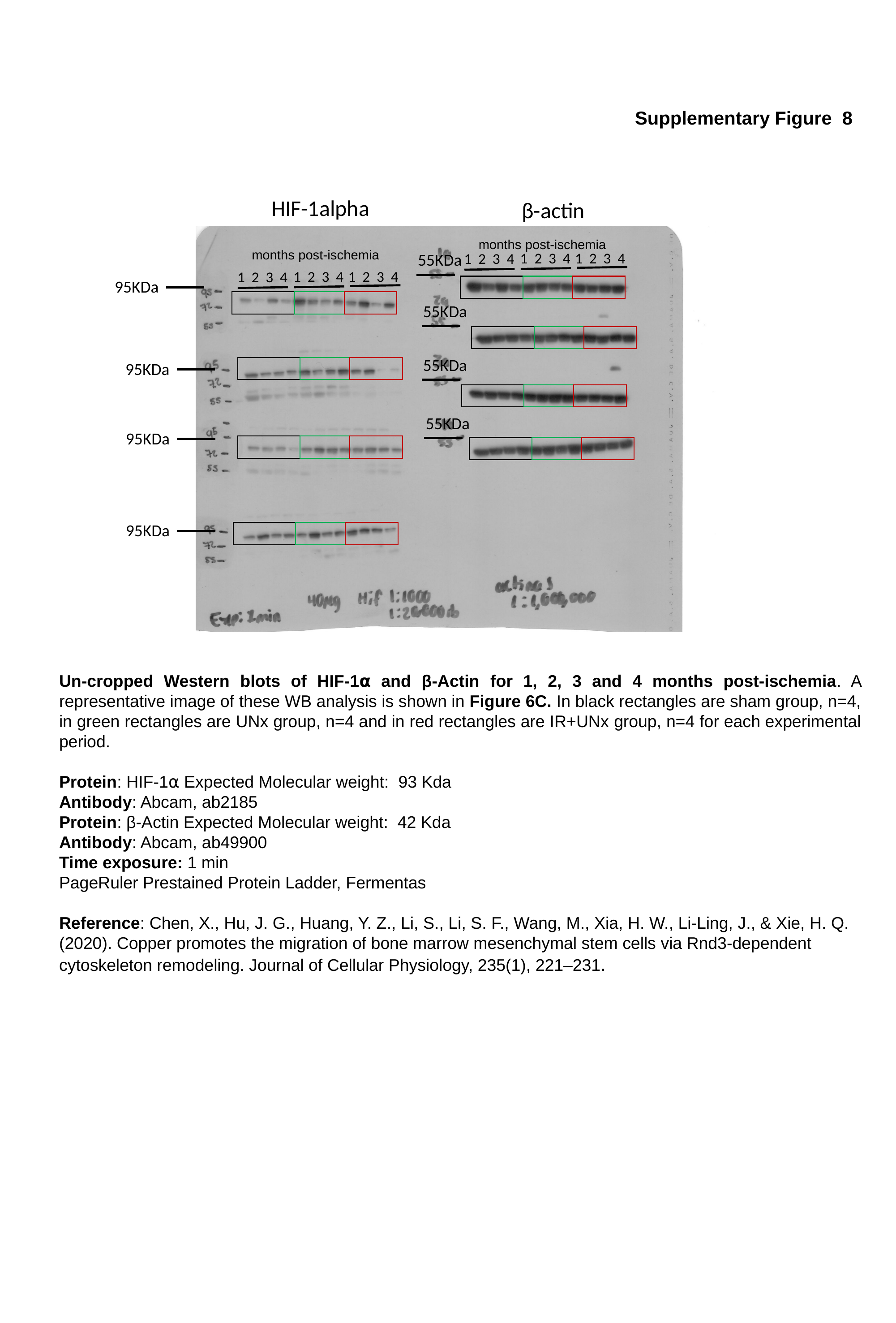

Supplementary Figure 8
HIF-1alpha
β-actin
months post-ischemia
months post-ischemia
1 2 3 4
1 2 3 4
1 2 3 4
55KDa
1 2 3 4
1 2 3 4
1 2 3 4
95KDa
55KDa
55KDa
95KDa
55KDa
95KDa
95KDa
Un-cropped Western blots of HIF-1⍺ and β-Actin for 1, 2, 3 and 4 months post-ischemia. A representative image of these WB analysis is shown in Figure 6C. In black rectangles are sham group, n=4, in green rectangles are UNx group, n=4 and in red rectangles are IR+UNx group, n=4 for each experimental period.
Protein: HIF-1⍺ Expected Molecular weight: 93 Kda
Antibody: Abcam, ab2185
Protein: β-Actin Expected Molecular weight: 42 Kda
Antibody: Abcam, ab49900
Time exposure: 1 min
PageRuler Prestained Protein Ladder, Fermentas
Reference: Chen, X., Hu, J. G., Huang, Y. Z., Li, S., Li, S. F., Wang, M., Xia, H. W., Li-Ling, J., & Xie, H. Q. (2020). Copper promotes the migration of bone marrow mesenchymal stem cells via Rnd3-dependent cytoskeleton remodeling. Journal of Cellular Physiology, 235(1), 221–231.

## Slide 10
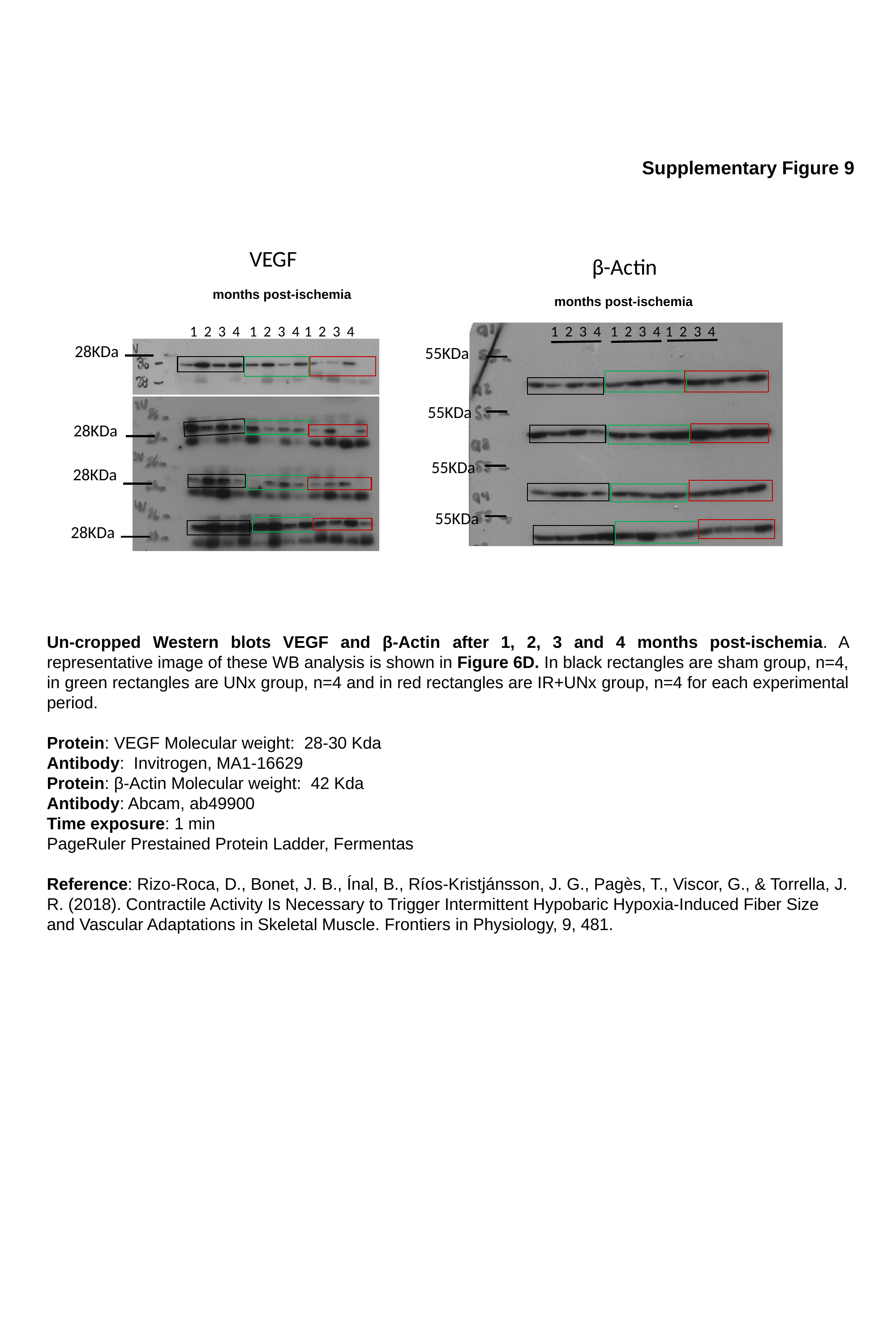

Supplementary Figure 9
VEGF
β-Actin
months post-ischemia
months post-ischemia
1 2 3 4
1 2 3 4
1 2 3 4
1 2 3 4
1 2 3 4
1 2 3 4
28KDa
55KDa
55KDa
28KDa
55KDa
28KDa
55KDa
28KDa
Un-cropped Western blots VEGF and β-Actin after 1, 2, 3 and 4 months post-ischemia. A representative image of these WB analysis is shown in Figure 6D. In black rectangles are sham group, n=4, in green rectangles are UNx group, n=4 and in red rectangles are IR+UNx group, n=4 for each experimental period.
Protein: VEGF Molecular weight: 28-30 Kda
Antibody: Invitrogen, MA1-16629
Protein: β-Actin Molecular weight: 42 Kda
Antibody: Abcam, ab49900
Time exposure: 1 min
PageRuler Prestained Protein Ladder, Fermentas
Reference: Rizo-Roca, D., Bonet, J. B., Ínal, B., Ríos-Kristjánsson, J. G., Pagès, T., Viscor, G., & Torrella, J. R. (2018). Contractile Activity Is Necessary to Trigger Intermittent Hypobaric Hypoxia-Induced Fiber Size and Vascular Adaptations in Skeletal Muscle. Frontiers in Physiology, 9, 481.
